# Development and Evaluation of Dissolving Microarray Patches for Co-administered and Repeated Intradermal Delivery of Long-acting Rilpivirine and Cabotegravir Nanosuspensions for Paediatric HIV Antiretroviral Therapy

**DOI:** 10.1007/s11095-022-03408-6

**Published:** 2022-10-12

**Authors:** Kurtis Moffatt, Ismaiel A. Tekko, Lalitkumar Vora, Fabiana Volpe-Zanutto, Aaron R. J. Hutton, Jessica Mistilis, Courtney Jarrahian, Nima Akhavein, Andrew D. Weber, Helen O. McCarthy, Ryan F. Donnelly

**Affiliations:** 1 School of Pharmacy, Queen’s University Belfast, Medical Biology Centre, 97 Lisburn Road, Belfast, BT9 7BL UK; 2 PATH, 2201 Westlake Avenue, Suite 200, Seattle, WA 98121 USA; 3 ViiV Healthcare, 1250 South Collegeville Rd, Collegeville, PA 19426 USA; 4 ViiV Healthcare, 410 Blackwell Street, Durham, 27701 NC UK

**Keywords:** AIDS, acquired immune deficiency syndrome, CAB, cabotegravir, HIV, human immunodeficiency virus, MAP, microarray patch, RPV, rilpivirine

## Abstract

**Purpose:**

Whilst significant progress has been made to defeat HIV infection, the efficacy of antiretroviral (ARV) therapy in the paediatric population is often hindered by poor adherence. Currently, two long-acting (LA) intramuscular injectable nanosuspensions of rilpivirine (RPV) and cabotegravir (CAB) are in clinical development for paediatric populations. However, administration requires access to healthcare resources, is painful, and can result in needle-stick injuries to the end user. To overcome these barriers, this proof-of-concept study was developed to evaluate the intradermal delivery of RPV LA and CAB LA via self-disabling dissolving microarray patches (MAPs).

**Methods:**

Dissolving MAPs of two conformations, a conventional pyramidal and a bilayer design, were formulated, with various nanosuspensions of RPV and CAB incorporated within the respective MAP matrix. MAPs were mechanically robust and were capable of penetrating *ex vivo* skin with intradermal ARV deposition.

**Results:**

In a single-dose *in vivo* study in rats, all ARV MAPs demonstrated sustained release profiles, with therapeutically relevant plasma concentrations of RPV and CAB detected to at least 63 and 28 d, respectively. In a multi-dose *in vivo* study, repeated MAP applications at 14-d intervals maintained therapeutically relevant plasma concentrations throughout the duration of the study.

**Conclusions:**

These results illustrate the potential of the platform to repeatedly maintain plasma concentrations for RPV and CAB. As such, these MAPs could represent a viable option to improve adherence in the paediatric population, one that is capable of being painlessly administered in the comfort of the patient’s own home on a biweekly or less frequent basis.

**Graphical abstract:**

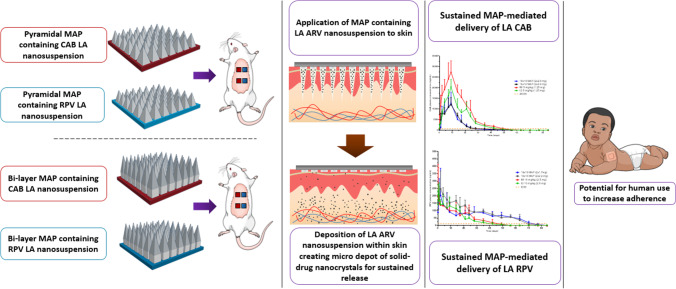

## Introduction

Antiretroviral (ARV) therapy has transformed the disease management for patients with HIV, severely reducing AIDS-related morbidity and mortality, evidenced by the average life expectancy of infected individuals now comparable to that of uninfected individuals [1]. In addition, when used consistently, pre-exposure prophylaxis (PrEP) has been shown to reduce HIV transmission rates in high-risk uninfected individuals by 92% when they consistently adhered to therapy [2]. However, despite such advancements in ARV therapy, HIV continues to remain a threat to global health.

At present, 37.6 million people worldwide are living with HIV, with approximately 1.7 million between 0 and 14 years of age [3]. The number of cases in the paediatric population has declined dramatically in recent years due to the continued progress in reducing mother-to-child transmission. Nevertheless, nearly 90% of the children infected with HIV live in sub-Saharan Africa, with limited access to ARV treatment [4]. Those who do receive ARV therapy in low-income countries often struggle with adherence due to high pill burdens, treatment costs, and limited access to healthcare facilities in rural areas. Furthermore, palatability of oral medication in the paediatric population, in addition to the rapid growth in early childhood, which necessitates tailored dosing, has resulted in suboptimal adherence. This contributes to therapeutic failure [5–7] and resultant emergence of drug-resistant viruses [8].

Efforts to enhance adherence make use of long-acting (LA) therapies capable of being administered on a monthly or less frequent basis, including LA injectable ARV agents, such as rilpivirine (RPV) [9] and cabotegravir (CAB) [10] nanosuspensions (NS), which release the drug into the systemic circulation for months following intramuscular (IM) administration. RPV is a non-nucleoside reverse transcriptase inhibitor approved as a bimonthly 900 mg prolonged-release suspension for injection for the treatment of HIV infection [11]. CAB is an integrase strand transfer inhibitor and structural analogue of dolutegravir approved as a bimonthly 600 mg prolonged-release suspension for IM administration [10]. Recently, a RPV/CAB drug combination (Cabenuva) has been approved by the US Food and Drug Administration[12]. This single-dose injectable suspension is the first LA HIV treatment regimen that has been approved for adolescents who are 12 years of age or older.

Despite reducing the pill burden through development of LA ARV injectables, such therapies are still subject to the drawbacks and limitations associated with standard hypodermic needle and syringe administration, such as pain, bleeding, and other injection site reactions, which may limit parents' acceptance within the paediatric population and hinder their widespread implementation [13, 14]. Furthermore, administration of ARVs via the IM route requires trained personnel and appropriate sharps disposal, which may limit access to this specific formulation which requires administration at 4- or 8-week intervals, particularly in low-income countries where the risk of misuse and inappropriate disposal of needles, leading to transmission of the bloodborne disease, is a significant issue [15–18]. Another drawback associated with ARV therapies is the need for cold chain storage – RPV LA NS requires refrigerated storage, which increases costs and may reduce or prevent access in low-income countries where cold chains cannot be monitored [19].

Microarray patches (MAPs) are a promising, minimally invasive method for intradermal (ID) administration of nanoformulated ARVs into the skin, thus circumventing the drawbacks associated with the use of a traditional hypodermic needle and syringe [20]. Dissolving MAPs are micron-scale devices, which can painlessly penetrate the stratum corneum, imbibe with interstitial fluid, and dissolve, releasing their therapeutic load. The ID route of administration has multiple advantages compared to delivery of ARVs via the IM route, such as the potential for application without the need for trained personnel [21] or the need for sharps disposal, as a dissolving MAP is self-disabling, with the potential for controlled release of ARV. Moreover, it may provide potential benefits compared to conventional oral ARV intake, avoiding gastrointestinal degradation and first-pass metabolism, resulting in dose sparing and possible improvement in patient adherence [22]. In addition, the dry-state nature of ARVs incorporated into dissolving MAPs could potentially overcome the need for cold chain storage and transport conditions [23]. The development of such a discreet, easily administered, and self-disabling delivery system for the delivery of ARVs within the paediatric population could therefore remove the adherence issues associated with daily oral ARV regimens. Previous studies involving RPV dissolving MAPs have shown early promise for the potential of this platform [24, 25], in line with existing HIV patient attitude surveys that have shown a positive enthusiasm for LA alternatives, particularly for the MAP approach [26].

In this proof-of-principle study, RPV and concentrated CAB LA NS were formulated into two different MAP designs. The ARV NS and MAPs were fully characterised, and their pharmacokinetic profiles were evaluated in Sprague Dawley rats for a period of 12 weeks following a single-dose administration. This approach indicates that RPV and CAB, administered by MAP, could be deposited intradermally as a micro-depot into the viable skin and that, as the nanoparticles dissolve in interstitial fluid, the drug would slowly be released into the systemic circulation, potentially maintaining therapeutic plasma levels for prolonged periods following the MAP removal [27]. Additionally, with the understanding that HIV is a chronic condition requiring lifelong treatment, in order to assess the repeated dosing potential of these dissolving MAPs, a multiple-application *in vivo* study evaluated the optimised MAPs’ ability to consistently maintain therapeutic plasma concentrations following repeated dosing.

The work presented here demonstrates, for the first time, the successful simultaneous delivery of an ARV regimen postulated for treatment of HIV infection, demonstrating the potential of this route of delivery and dissolving MAP system to achieve a sustained release of two or more ARV agents in the paediatric population. Consequently, this delivery approach of combining both NS and MAPs, represents an opportunity to overcome the current requirement for adherence to oral multi-drug ARV regimens and further circumvents some of the problems associated with hypodermic needle delivery for the administration of IM LA ARV NS.

## Materials and Methods

### Materials

RPV free-base powder and RPV LA NS (RPV LA 300 mg/mL) were supplied by Janssen Pharmaceutica (Beerse, Belgium). CAB free acid and CAB LA NS (CAB LA 200 mg/mL) were supplied by ViiV Healthcare Ltd. (Research Triangle Park, North Carolina, United States). Poly(vinyl alcohol) (PVA) 9–10 kDa (80% hydrolysed), PVA 31–50 kDa, trifluoroacetic acid (TFA) (> 99%), and D-mannitol (> 98%) were purchased from Sigma-Aldrich (Gillingham, Dorset, United Kingdom). Poly(vinylpyrrolidone) (PVP) 58 kDa, PVP 360 kDa, were provided by Ashland (Kidderminster, United Kingdom). Dimethyl sulfoxide (DMSO) and glycerol bidistilled, AnalaR NORMAPUR® analytical reagent (99.5%) were purchased from VWR International (Leicestershire, United Kingdom). Pluronic® F-108 (PF108) was purchased from BASF SE (Ludwigshafen, Germany). Poly(lactic acid) Silver Metallic (9088) was supplied by Ultimaker (Geldemalsen, Netherlands). Acetonitrile LiChrosolv® hypergrade was purchased from Merck (Darmstadt, Germany). Ultrapure water was obtained from a water purification system, the Elga PURELAB DV-25, from Veolia Water Systems (Celbridge, Ireland). All other chemicals were of analytical reagent grade and purchased from standard industrial suppliers.

### Methods

#### Formulation, Optimisation, and Post-Production Processing of ARV NS

##### Anti-solvent precipitation-ultrasonication of novel RPV NS

Novel RPV NS were prepared using an anti-solvent precipitation ultrasonication technique, as described previously [28] but with slight modifications. Initially, 90 mg of RPV free-base was completely dissolved in 1 mL of DMSO and vortex-mixed to ensure complete dissolution in preparation for the solvent phase. Meanwhile, in a separate vial, the antisolvent phase was prepared by dispersing Pluronic F-108 in water to a concentration of 0.2% w/v. At a temperature of 2°C to 8°C, maintained by an ice-water bath, 1 mL of solvent phase was injected drop-wise into 4 mL of antisolvent phase under sonication using an ultrasonic probe (Fisher Scientific Co. Ltd., Pittsburgh, Pennsylvania, United States) at an ultrasound frequency of 20 kHz for 10 min, with episodic bursts and breaks in ultrasound (10 s pulse on and 5 s pulse off) to form the NS. In a synergetic attempt to ensure complete removal of excess DMSO and concentrate the RPV NS dosage form, resultant formulations were lyophilised. Prior to the lyophilisation process, mannitol 5% w/v was added to the RPV NS as a cryoprotectant. The mixture was pre-frozen at –80°C for 2 h and then transferred to a VirTis AdVantage® Bench Top Pro Freeze Dryer System (SP Scientific, Warminster, Pennsylvania, United States) for a 26-h cycle to produce dry powder NS. Mean particle size, polydispersity index (PDI), zeta potential, and RPV concentration were recorded pre- and post-lyophilisation.

##### Post-Production Concentration Enhancement of CAB LA NS

CAB LA NS were supplied by ViiV Healthcare Ltd., with full excipient and formulation details listed. The total mass of the contents of each vial was 2.145 ± 0.04 g, equating to 250 mg/g of CAB and excipients. Excipients (mannitol, polysorbate 20, and polyethylene glycol 3350) accounted for 50 mg/g; therefore, CAB content accounted for approximately 200 mg/g of the NS within each vial. In an attempt to maximise eventual CAB drug content localised within the dissolving MAP tips, using a method described previously [29], removal of the excipient containing supernatant via needle and syringe was investigated to concentrate the CAB LA NS dosage form, whilst having minimal effect on particle characteristics. A measurement of mass loss was performed to ensure that the supernatant removed was reproducible each time. Mean particle size, PDI, zeta potential, and CAB concentration were all recorded after the concentration enhancement process.

#### Characterisation of ARV NS

Mean particle size, PDI, and zeta potential of the ARV NS were measured via dynamic light scattering using a NanoBrook Omni particle sizer (Brookhaven Instruments Corporation, New York, New York, United States). NS in either the aqueous or the lyophilised form were initially diluted in water and stabilised at 25°C prior to analysis. The determination of NS drug content was performed using high-performance liquid chromatography (HPLC), as described below (Sect. Pharmaceutical Analysis of RPV and CAB).

Various techniques were used to identify potential physical or chemical interactions in the novel RPV NS. Attenuated total reflectance–Fourier transform infrared (ATR-FTIR) spectral studies were conducted using a FTIR spectrophotometer, the Accutrac™ FT/IR-4100 Series (Jasco Corporation, Easton, Maryland, United States). Differential scanning calorimetry (DSC) studies were conducted using a differential scanning calorimeter, the DSC Q100 (TA Instruments, New Castle, Delaware, United States). Powder X-ray diffraction (P-XRD) studies were conducted using the P-XRD Diffractometer MiniFlex II (Rigaku Corporation, Kent, United Kingdom). Lastly, thermogravimetric analysis was used to ensure complete removal of excess solvent through lyophilisation processes by a thermogravimetric analyser, the Thermal Advantage Model Q500 (TA Instruments).

#### *In Vitro* RPV Drug Release Study

A dialysis membrane method was utilised to investigate the in vitro rate of release of RPV from the novel RPV NS using a modification of a previously established method [30]. The RPV base and RPV NS dispersion were placed in Spectra/Por®7, 12 000–14 000 MWCO (molecular weight cut-off) dialysis membrane (Spectrum Medical Industries Inc., Laguna Hills, California, United States) and suspended in 100 ml of phosphate-buffered saline (PBS):methanol (20:80, v/v) release medium in an orbital shaker, the Infors-HT Unitron Incubator Shaker (Analab, Nottingham, United Kingdom) at 60 rpm and 37.5°C. Additionally, a 1 mL aliquot of sample was taken at predetermined time intervals and replaced by 1 mL fresh release medium. To quantify the amount of drug released from RPV NS, the samples were then quantified by HPLC. The cumulative RPV released was then fitted.

#### Evaluation of Novel Lyophilised RPV NS Short-Term Stability

Novel RPV NS were evaluated for their short-term stability at room temperature (19°C). RPV NS in lyophilised form were stored in sealed amber vials, protected from light, to evaluate the sole effect of temperature upon particle characteristics and drug content recovery. Mean particle size and RPV concentration were recorded at predetermined time intervals (0 d, 1 d, 2 d, 3 d, 7 d, 14 d, 21 d, and 28 d) across a 28-d period.

#### Evaluation of the Effect of Polymer and MAP Formulation Upon Particle Size and PDI of ARV NS

Aqueous polymeric-NS blends and dry optimised MAP formulations containing ARV NS were dispersed in water. Following this, the mean particle size and PDI were determined by dynamic light scattering and were compared to that of the original NS particle properties.

#### Fabrication of Two-Layered Dissolving 19 × 19 MAPs Containing ARV NS

**Fig. 1:**
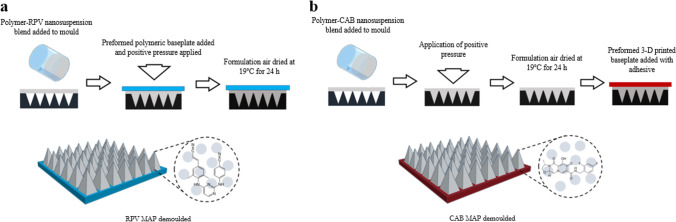
Diagrammatic representation of fabrication process of pyramidal 19 × 19 ARV MAPs, displaying (**a**) fabrication process of 19 × 19 RPV MAPs and (**b**) 19 × 19 CAB MAPs.

Various aqueous gel-based MAP formulations were prepared using biocompatible polymers at different concentrations – namely, PVA (9–10 kDa) and PVP (58 kDa). The MAP formulation compositions are outlined in Tables I and II for RPV and CAB, respectively. The process for fabrication of both RPV and CAB MAPs is illustrated schematically in Fig. 1. Initially, the aqueous formulations of selected polymers were added to the lyophilised RPV or aqueous concentration-enhanced NS CAB, and the polymer-NS blend was mixed via Speedmixer™ DAC 150 FVZ-k (FlackTek Inc., Landrum, South Carolina, United States) at 3500 rpm for 3 min to achieve a homogenous blend. The resulting formulation was then poured (40 mg) onto injection-moulded silicone micromoulds (LTS Lohmann Therapie-Systeme, Andernach, Germany), the template consisting of 361 pyramidal needles (19 × 19 needle density; 0.49 cm^2^, 500 μm height; 300 μm base width; and 30 μm interspacing). At this stage, for RPV MAPs only, a pre-cast dry baseplate (with an area of 1 cm^2^), fabricated from aqueous gels of 20.0% w/w PVP 360 kDa and 1.5% w/w glycerol, was placed on top of the MAP formulations. All moulds were then placed within a positive pressure chamber, and a pressure of 3 to 4 bar was applied for 15 min. Finally, the MAPs were dried at room temperature for 24 h and then removed from the moulds. At this stage, for CAB MAPs only, a preformed 3-D printed poly(lactic acid) baseplate (with an area of 1 cm^2^) was applied onto the back of the dried formulation with double-sided tape on the downward-facing side to adhere to the formulation. The timing of the baseplates addition and composition was previously optimised, dependent upon on the viscosity of the MAP formulation and the requirement for air-drying; as such, RPV MAP formulations, being more viscous, were added before drying, and CAB MAPs, being more aqueous, were added after air-drying. The morphology of the MAPs was observed using a digital light microscope (Leica Microsystems, Milton Keynes, United Kingdom) and scanning electron microscope (SEM) TM3030 (Hitachi, Krefeld, Germany).

**Table I:** Compositions of Various Formulations Used to Prepare Pyramidal 19 × 19 RPV MAPs

Formulation code	% (w/w) lyophilised novel RPV NS	Equivalent % RPV content	% (w/w) PVA 10 kDa	% (w/w) PVP 58 kDa
RPV 1.1	28.2	20.0	15	-
RPV 1.2	35.2	25.0	20	-
RPV 1.3	38.7	27.5	20	-
RPV 1.4	42.3	30.0	20	-
RPV 1.5	45.8	32.5	25	-
RPV 1.6	45.8	32.5	20	5

**Table II:** Compositions of Various Formulations Used to Prepare Pyramidal 19 × 19 CAB MAPs

Formulation code	% (w/w) concentration-enhanced CAB LA NS	% (w/w) PVA 10 kDa	% (w/w) PVP 58 kDa
CAB 1.1	70	15.0	-
CAB 1.2	40	15.0	15.0
CAB 1.3	50	12.5	12.5
CAB 1.4	60	10.0	10.0
CAB 1.5	70	07.5	07.5
CAB 1.6	70	10.0	05.0

#### Fabrication of Bilayer Dissolving 16 × 16 MAPs Containing ARV NS

**Fig. 2:**
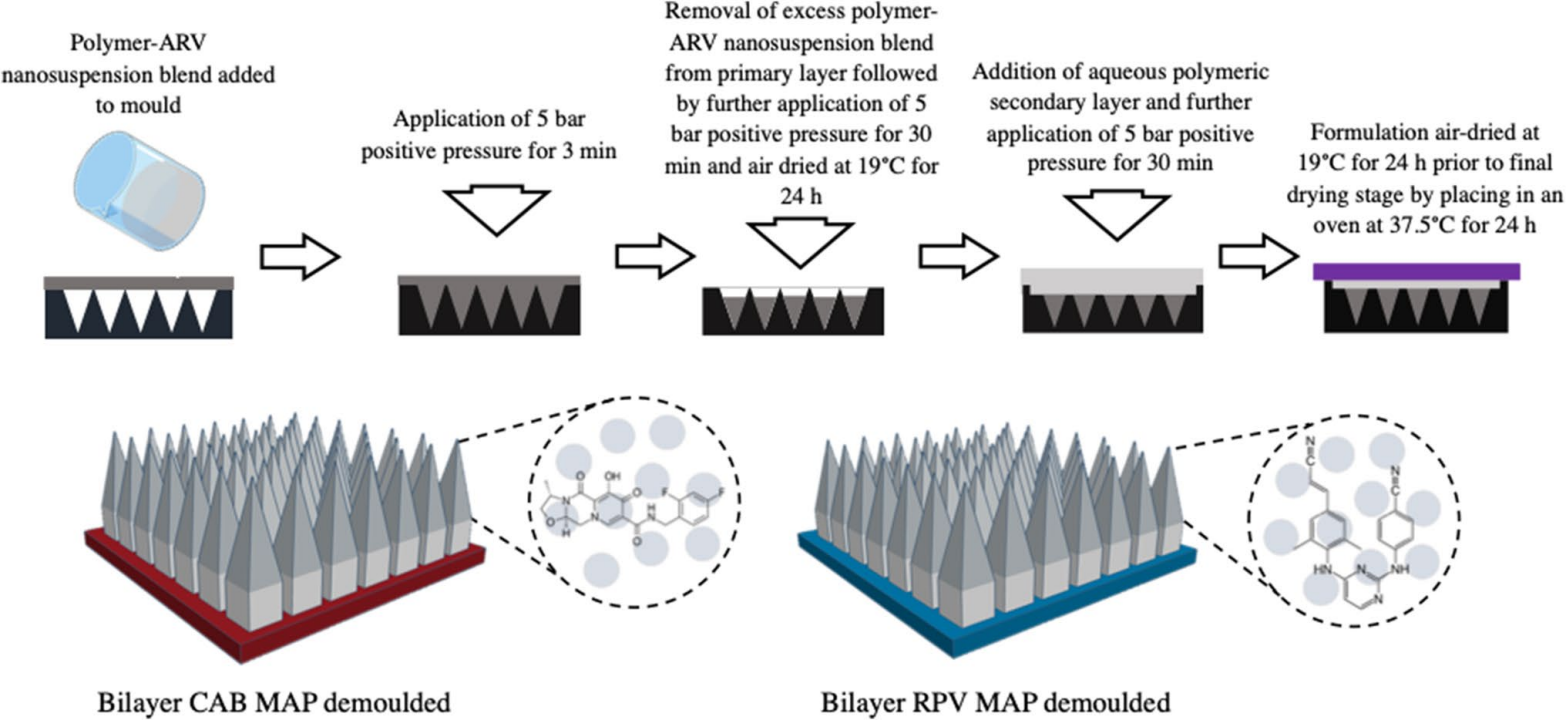
Diagrammatic representation of fabrication process of bilayer 16 × 16 ARV MAPs.

Bilayer 16 × 16 MAP moulds, as detailed by Tekko *et al.* [29] were used with the intended purpose to localise and increase ARV NS loading within the MAP tips and further enhance skin penetration by taking into consideration tip shape and array density. Similar to the pyramidal 19 × 19 MAP manufacture, various aqueous gel-based MAP formulations for the pyramidal tip were prepared using biocompatible polymers at different concentrations. The formulation compositions are outlined in Table III. As illustrated in Fig. 2, the manufacturing process for 16 × 16 MAPs includes an additional step to that of 19 × 19 MAPs (Fig. 1). Here, an additional drug-free aqueous polymeric secondary layer is added to concentrate the ARV NS into the needle tips.

**Table III:** Composition of Various Formulations Used to Prepare bilayer RPV and CAB 16 × 16 MAPs

ARV	Formulation code	% (w/w) novel lyophilised RPV NS	% (w/w) aqueous RPV LA NS	% (w/w) concentration-enhanced CAB LA NS	% PVA 10 kDa	% PVP 58 kDa
RPV	RPV 2.1	35.2	-	-	20	-
	RPV 2.2	-	70	-	15	-
	RPV 2.3	-	60	-	20	20
CAB	CAB 2.1	-	-	70	15	5
	CAB 2.2	-	-	60	20	20

Initially, the aqueous formulations of selected polymers were added to the lyophilised form of the novel RPV NS, the aqueous forms of proprietary RPV LA, or the concentration-enhanced CAB LA NS, and the polymer-NS blend then mixed via the Speedmixer DAC 150 FVZ-k at 3500 rpm for 3 min to achieve a homogenous blend. The resulting formulation was then poured (40 mg) onto laser-engineered silicone micromoulds, the template consisting of 256 pyramidal needles (16 × 16 needle density, 0.49 cm^2^, 850 μm height [600 μm pyramidal tip, 250 μm base column], 300 μm column width, and 100 μm interspacing). The mould was then placed on a flat surface within a pressure chamber, and positive pressure of 5 bar was applied for 3 min to ensure adequate initial filling of the MAP tip cavities. Moulds were then removed from the pressure chamber, and excess formulation was removed from the surface by the edge of a spatula. The mould was then once again placed into the pressure chamber, and a positive pressure of 5 bar was applied for a further 30 min. Upon removal from the chamber, the MAPs were then air-dried at room temperature for 24 h on a levelled surface. Following initial drying, an aqueous polymeric blend consisting of 50% w/w PVA 31–50 kDa (30% w/w) and 50% w/w PVP 58 kDa (40% w/w) was then poured slowly onto the mould to form a baseplate and achieve the drug-free second layer in the MAP formulation. Moulds were then placed on a flat surface within the pressure chamber, and a positive pressure of 5 bar was applied for 30 min, slowly increasing the pressure incrementally. The formulation was then air-dried at room temperature for a further 24 h on a levelled surface. After the second drying stage, moulds were then placed in an oven at 37°C for a final 24 h to complete drying. Following the second drying step, completely formed MAPs were removed from the moulds. The morphology of the MAPs was then observed, as detailed previously for the pyramidal 19 × 19 MAPs.

#### Characterisation of Mechanical and Insertion Properties of ARV MAPs

The mechanical strength of the MAPs was investigated using a TA-TX2 Texture Analyser (Stable Microsystem, Surrey, United Kingdom) in compression mode to evaluate the mechanical strength of the MAPs, as previously described [31]. To evaluate the insertion capability of the MAPs, Parafilm® M (Amcor Flexibles North America Inc., Zurich, Switzerland) was used as an artificial skin simulant model, as detailed previously [32, 33].

#### Evaluation of ARV Drug Content Localised Within MAP Tips

##### Calculation of Theoretical ARV Drug Content Localised Within the Pyramidal 19 × 19 MAP Tips

As ARV was present in both the baseplate and the needles themselves, mathematical modelling was necessary to determine the theoretical ARV drug content localised within the pyramidal 19 × 19 MAP tips. Initially, the density of each formulation was calculated.by preparing rectangular films of each formulation. After drying, the dimensions and the mass of the films were determined and used to calculate the density of the films using Eq. (1):

1 $$\uprho = \frac{mg}{SX}$$

where ρ is the density of the dry formulation, mg is the mass of the film, X is the mean thickness of the film, and S is the cross-sectional area of the film.

To calculate the ARV amount (mg) in the needle tips, Eq. (2) was used [27]:

2 $$ARV\;\;in\;\;the\;\;needle\;\;tips=N\bullet\frac{\left(h\bullet b^2\right)\bullet\rho\bullet\lbrack ARV\rbrack}3$$

where N is the number of needle tips (361 tips), h is the height of the tips (600 µm), b^2^ is the width of base of the tips (300^2^ µm), ρ is the density of the dry formulation, and [ARV] is the concentration of ARV in dry formulation (expressed in mg ARV/mg material).

##### Determination of drug content localised within the bilayer 16 × 16 MAPs

Due to the bilayer design for the 16 × 16 MAPs, the ARV drug was localised solely within the pyramidal tips, separated from the baseplate by a drug-free polymeric base column. Therefore, the total drug content in the tips could be accurately quantified by pharmaceutical analysis via HPLC. Five replicates of each ARV LA MAP were placed into separate 2 mL Eppendorf® tubes (Eppendorf UK Ltd., Stevenage, United Kingdom) and dissolved in 2 mL of deionised water. The samples were then vortex-mixed for 1 h to ensure complete dissolution of the MAP. Following this, 10 μL of solution was extracted and added to a further aliquot of 190 μL deionised water and vortexed for a further 10 min. This solution was then directly added to 800 μL of acetonitrile and centrifuged at 14 000 rpm for 10 min. An aliquot of 100 μL supernatant was removed, and the ARV content was determined by HPLC.

#### Ex Vivo In-Skin Dissolution and Drug deposItion Studies

The dissolution rate and drug deposition of the ARV LA MAPs were investigated in full-thickness-excised neonatal porcine skin collected from stillborn piglets. Skin samples were carefully shaved using a disposable razor and pre-equilibrated in PBS (pH 7.4) for 30 min before being mounted onto a polystyrene foam block coated with PBS-soaked absorbent paper. MAPs were inserted into the skin with a pressure of 32 N/MAP over 30 s using a TA.XTPlus Texture Analyser (Stable Micro Systems, Surrey, United Kingdom) [32]. A cylindrical steel weight of 5.0 g was then placed on the back of the MAPs to ensure they remained *in situ* for the duration of the experiment, and the skin setup was then incubated at 33°C for 24 h to mimic the average skin temperature of *in vivo* conditions. MAPs were removed at predetermined time points of 1, 5, and 24 h, representative of the initial blood sampling time points in *in vivo* studies, and the skin was washed thoroughly with deionised water to remove residual formulation remaining on the skin surface. Subsequently, tissue samples were taken from the portion of the skin where the ARV LA MAP had been inserted, obtained via biopsy punch (12 mm diameter) (Stiefel, Middlesex, United Kingdom). The excised tissue was then placed into a 2 mL Eppendorf tube. To extract the drug from the tissue, 0.5 mL of deionised water was added, and each sample was homogenised with two stainless steel beads for 10 min at 50 Hz using a TissueLyser LT (Qiagen Ltd., Manchester, United Kingdom). A further 1 mL of acetonitrile was then added to the sample and subjected to a further cycle of homogenisation to complete analyte extraction from the skin. The samples were then centrifuged at 14 000 rpm for 10 min. The supernatant was then collected and analysed using HPLC. A needle-free patch was used as a comparator control in the deposition studies. Upon removal from the skin, MAPs and skin were digitally imaged by light microscope (Leica EZ4 D digital light microscope, Leica Microsystems, Milton Keynes, United Kingdom), and observations of dissolution rate and visible skin deposition were noted.

#### *In Vivo* Studies

Ethical permission for all *in vivo* experiments was obtained from the committee of the Queen's University Belfast, Biological Services Unit. All researchers held Personal Licences (PILs 1747, 1889, and 1892) from the UK Home Office, and work was conducted under Project Licence 2794. All experiments were conducted according to the policy of the Federation of European Laboratory Animal Science Associations and the European Convention for the Protection of Vertebrate Animals used for Experimental and other Scientific Purposes, with the implementation of the principles of the 3 R’s – replacement, reduction, and refinement. Two experimental designs were devised and conducted in the *in vivo* studies: Phase 1, a single-dose administration study for pharmacokinetic evaluation (84 d), and Phase 2, a multiple repeated-dose administration study for reproducibility of maintenance dosing evaluation (42 d), as reported in more detail in the respective section below. In all cohorts, both RPV and CAB were administered simultaneously within the same animal model.

Experiments were conducted using female Sprague–Dawley rats aged between 10 and 13 weeks upon commencement of the study and aged between 22 and 25 weeks and 16 and 18 weeks upon completion of Phase 1 and Phase 2, respectively. Rats initially weighed 219.88 ± 18.44 g and 220.83 ± 15.82 g for Phase 1 and Phase 2, respectively, and all rats were acclimatised to conditions within the Biological Services Unit for a minimum of 7 d prior to commencement of the experiments.

##### General Animal Procedures

**Fig. 3:**
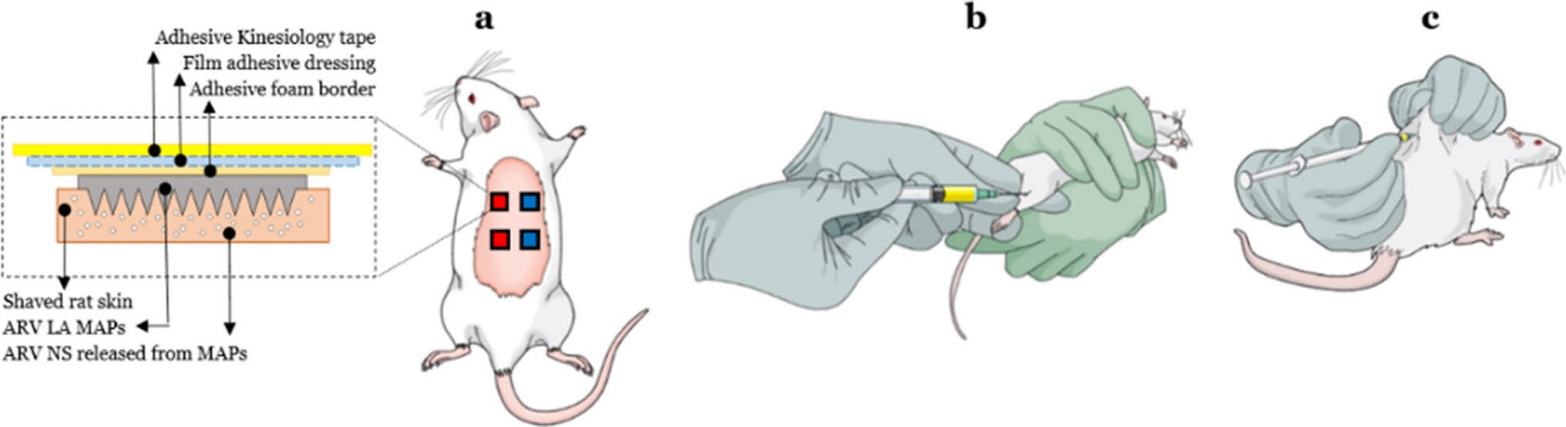
Schematic representations of the rat cohorts in the *in vivo* delivery of RPV and CAB. (**a**) MAP-mediated ID delivery cohort of four MAP arrays was used, with blue squares representative of RPV MAPs and red squares representative of CAB MAPs. (**b**) In the first control cohort, rats received an IM injection of RPV LA into the right thigh and IM injection of CAB LA into the left thigh. (**c**) Similarly, in the second control cohort, rats received an ID injection of RPV LA into the right-hand side and an ID injection of CAB LA into the left-hand side of their backs.

For application of MAPs, rats were initially anaesthetised using isoflurane (2% to 4% in oxygen), and the hair from their backs was shaved using an electric razor, and Veet® Sensitive Skin hair removal cream (Reckitt Benckiser Group, Slough, United Kingdom) was applied for 6 min before removal using absorbent paper and deionised water. The rats were observed to ensure no adverse effects developed and then left to recover for 24 h. In all the MAP cohorts, the rats received four MAPs, applied to hair-free, clean, dry skin on their backs. In all cases, on the right-hand side two RPV MAPs were applied, and on the left-hand side two CAB MAPs were applied. The ARV MAPs were manually inserted into the skin by application of firm finger pressure for 30 s, followed by the addition of a foam adhesive border (Microfoam® tape, 3 M, St. Paul, Minnesota, United States) to assist in MAP retention in the skin. To ensure MAPs remained securely in place, an adhesive film (Tegaderm™ film, 3 M) was placed on top of the MAPs and the surrounding skin. A final layer of adhesive kinesiology tape (Proworks Corporation, Corvallis, Oregon, United States) was wrapped around the rats’ backs and torsos to ensure the rats did not displace the MAPs from their skin overnight (Fig. 3). The rats were then individually housed for 24 h following MAP application, prior to subsequent MAP removal.

Blood samples were collected by lateral tail bleeds access using a 23G butterfly needle at predetermined time points throughout the study. Briefly, the rats were individually placed into a heat box at < 39°C for a maximum of 10 min, following which the animals were restrained using a surgical cloth. The tail was gentled bathed with ethanol (70% v/v) to prevent infection, following which the lateral tail vein was identified and needle inserted approximately one-third from the tip of the tail. A maximum of 200 μL of blood was collected into heparinised tubes. In keeping with the University’s Institutional Project Licence, rats were not subject to more than two bleeds in one 24-h period, and no more than ten bleeds were collected in each month of experimentation. Furthermore, rats were not exposed to more than two heat cycles in a 24-h period, in accordance with the Queen’s University Biological Sciences Laboratory policy. After collection of blood, plasma separation was performed by centrifugation of the blood at 5500 rpm for 15 min at 4°C. Plasma was then collected into a non-heparinised 1.5 mL Eppendorf tube and stored at –80°C until analyte extraction and analysis.

##### Phase 1: Evaluation of Plasma Pharmacokinetics of RPV and CAB Following a Single MAP Application

The initial (Phase 1) *in vivo* study evaluated plasma pharmacokinetics of both ARVs following a single MAP dose administration over a period of 84 d, to allow for complete elimination of the respective ARV. The animals were separated into four cohorts (*n* = 6 per cohort) (Table IV). These consisted of two MAP cohorts, each exploring two different MAP conformations. The first MAP cohort investigated the 19 × 19 pyramidal array design (containing the optimised RPV NS formulation and the concentration-enhanced CAB LA NS formulation), and the second MAP cohort examined the 16 × 16 bilayer cuboidal base-pyramidal tip array design (containing RPV LA NS and the concentration-enhanced CAB LA NS). In each cohort, rats were treated with four MAPs (two RPV and two CAB MAP arrays) (Fig. 3a). The third and fourth cohorts represent the controls by IM and ID administration, respectively, of both proprietary ARV LA NS. The third cohort received an IM dose of 50 μL of RPV LA NS (10 mg/kg) into the right thigh and an IM dose of 50 μL of CAB LA NS (5 mg/kg) into the left thigh (Fig. 3b). The fourth cohort similarly received the same doses of RPV LA and CAB LA NS via ID injection into the skin on the right and left sides of the rats’ backs, respectively (Fig. 3c). The cohort design within the single application *in vivo* experiment is summarised in Table IV.

**Table IV:** *In Vivo* Study Plan Showing the Treatment Cohort Design for the Phase 1 Pharmacokinetic Experiment

	Treatment cohort			
	1 19 × 19 MAP	2 16 × 16 MAP	3 IM	4 ID
RPV	1 MAP = 1.7 mg (2 MAP = 3.4 mg per rat)	1 MAP = 2.2 mg (2 MAP = 4.4 mg per rat)	10 mg/kg (2.5 mg per rat)	10 mg/kg (2.5 mg per rat)
CAB	1 MAP = 2.8 mg (2 MAP = 5.7 mg per rat)	1 MAP = 2.8 mg (2 MAP = 5.7 mg per rat)	5 mg/kg (1.25 mg per rat)	5 mg/kg (1.25 mg per rat)

##### Phase 2: Evaluation of Plasma Pharmacokinetics of RPV and CAB Following Multiple Repeated MAP Applications

The second (Phase 2) *in vivo* study evaluated the ability of MAPs to reproducibly maintain therapeutic plasma levels following multiple repeated MAP applications over a period of 42 d and further examined the effect of an IM loading dose of ARV LA NS. This experiment was set up to loosely correlate to the doses used in IM LA ARV clinical trials and to that of the newly marketed Cabenuva LA injection [34].

The dosing interval was further established by taking into account the difference in pharmacokinetics between species and individual ARV pharmacokinetics observed in Phase 1 *in vivo* studies, whilst also carefully considering the welfare of the animals and blood sampling restrictions. As such, a dosing interval of 14 d was selected as most appropriate, with a total of three dose administrations across the duration of the experiment.

The animals were separated into two cohorts (*n* = 6 per cohort). The first cohort involved initial administration of an IM loading dose of each ARV LA NS, followed by two repeat applications of dissolving ARV MAPs every 14 d. The second cohort involved solely three repeat applications of dissolving ARV MAPs at the same time intervals as the initial cohort.

The rats’ skin was prepared, as previously described, each time for MAP applications, and similarly, IM injection was administered into the thighs of the rat as a loading dose in the first cohort, as previously detailed. For MAP applications, four 16 × 16 bilayered MAPs (two RPV and two CAB MAPs) were applied to each rat, as detailed previously, at the designated time points for cohort 1 (14 and 28 d*)* and cohort 2 (0, 14, and 28 d). The cohort design and respective quantity of ARV dose delivered within the multiple-application *in vivo* experiment are summarised in Table V.

**Table V:** *In Vivo* Study Plan Showing the Treatment Cohort Design for the Phase 2 Multiple-Application Experiments

		Time point (d)		
		0	14	28
Treatment cohort		Loading Dose Administration	Second Dose Administration	Third Dose Administration
1: IM then MAP	RPV	20 mg/kg (5.0 mg per rat)	2 MAP (4.4 mg per rat)	2 MAP (4.4 mg per rat)
	CAB	10 mg/kg (2.5 mg per rat)	2 MAP (5.7 mg per rat)	2 MAP (5.7 mg per rat)
2: MAP only	RPV	2 MAP (4.4 mg per rat)	2 MAP (4.4 mg per rat)	2 MAP (4.4 mg per rat)
	CAB	2 MAP (5.7 mg per rat)	2 MAP (5.7 mg per rat)	2 MAP (5.7 mg per rat)

##### Plasma Sample Preparation and Analyte Extraction

For plasma samples, RPV and CAB were simultaneously extracted using acetonitrile with a one-step protein precipitation method. Acetonitrile (900 μL) was added to an aliquot of 100 μL rat plasma and vortexed for 15 min in a 1.5 mL centrifuge tube. The samples were then centrifuged at 14 000 rpm at 4°C for 15 min. The supernatant was removed and placed in a disposable glass culture tube. The sample extract was then evaporated under a stream of nitrogen at 35°C for 40 min using a Zymark TurboVap® LV Evaporator Workstation (McKinley Scientific, Sparta, New Jersey, United States). The residue was then reconstituted in 100 μL acetonitrile, and this solution was then vortex-mixed for 30 s, sonicated for 5 min, and centrifuged at 14 000 rpm for 15 min at room temperature. The reconstituted solution was then collected in a 500 μL Eppendorf tube and centrifuged at 14 000 rpm for 10 min at room temperature by an Eppendorf MiniSpin® centrifuge (Eppendorf UK Ltd., Stevenage, United Kingdom). The supernatant was then transferred into an Agilent® HPLC vial containing an Agilent® 250 µL vial insert (Agilent Technologies Inc., Santa Clara, California, United States), and both ARVs were quantified at the same time using HPLC–mass spectrometry.

#### Calculation of Basic Pharmacokinetic Parameters and Relative Bioavailability (F)

Basic non-compartmental analysis of the plasma pharmacokinetic profiles was conducted by PK Solver software [35]. The curve of drug concentration *versus* time profiles was plotted for both ARVs for each of the MAP designs (19 × 19 and 16 × 16) in the single-MAP-application *in vivo* study. The maximum drug concentration (C_max_), the time of maximum concentration (T_max_), and the area under the curve (AUC) for the drug concentration time from time zero (t = 0) to the last experimental time point (AUC_t0-*final*_) were all calculated, with final t = 84 (AUC_t0–84_).

The relative plasma bioavailabilities (F_MAP_) of RPV and CAB after ID delivery of NS-loaded MAPs following a single MAP application were compared directly to IM administration and calculated using Eq. (1).

1 $${F}_{MAP}={F}_{IM}(\frac{{AUC}_{t0-84\left(MAP\right)}x {dose}_{IM}}{{AUC}_{t0-84(IM)}x {dose}_{MAP}})$$

where AUC_MAP_ is the AUC of the plasma from NS-loaded MAPs administration, and AUC_IM_ is the AUC of plasma from IM administration of the proprietary LA NS.

Similarly, for the multiple-MAP-application *in vivo* study, non-compartmental analysis was conducted calculating the same parameters as previously described. Additionally, the minimum drug concentration reached prior to administration of a sequential dose (C_min_) was calculated. Furthermore, the drug concentration time curve from time zero (t = 0) to the last experimental time point (t = 42) (AUC_t0–42_) was adjusted for the second study to take into consideration the experimental endpoint.

#### Extrapolation to Human Dose and Translation to Patch Size

Rudimentary estimations of human dose for both ARVs and the two respective MAP designs were calculated based upon the therapeutic plasma trough levels observed following a single dose of 600 mg RPV LA IM and 400 mg CAB LA IM after 28 d in humans. This was selected as this is the monthly IM dosing regimen in clinical development [36]. Given that the initial pharmacokinetic study evaluated a single-dose ARV LA MAP and ARV LA IM, the exposure achieved after single-dose administration of RPV LA and CAB LA IM in humans was selected as opposed to trough concentrations achieved at steady state. Therapeutically relevant concentrations for RPV and CAB in humans should be maintained above the 90% inhibition concentration (IC_90_) values of 12 ng/mL and 166 ng/mL, respectively; however, the 4xIC_90_ is preferred for CAB as a more accurate extrapolation from non-human models (664 ng/mL) [37]. Using the mean concentration–time data, the F ratios of exposures after adjusting for differences in doses administered between MAP and IM administration were estimated, as detailed in the previous section. Considering this, alongside the mean plasma concentrations observed at 28 d in the initial *in vivo* study, cautious extrapolations to human dose were calculated and then further translated to a MAP therapeutic area size for each ARV and MAP design [36].

#### Pharmaceutical Analysis of RPV and CAB

The individual quantification of RPV and CAB from *in vitro* and *ex vivo* samples was performed using HPLC, by UV detection (1200 Infinity, Agilent Technologies Inc.). The chromatographic separation for RPV was carried out using a SphereClone™ (Phenomenex, Torrance, California, United States) 5μ (ODS-1) column (150 mm × 4.60 mm internal diameter, 5 μm packing) and, for CAB, an Inertsil® (GL Sciences Inc., Torrance, California, United States) ODS-3 C18 column, (250 mm × 4.60 mm internal diameter, 5 μm packing). The chromatographic conditions for each respective method are reported in Table I. Prior to use, mobile phases were degassed via filtration and sonication. Agilent Chemstation® Software B.02.01 was used for chromatographic analysis. The limits of detection (LoD) and quantification (LoQ) for each ARV and its respective method are further detailed in Table VI. All analytical methods were validated in accordance with the Q2(R1) guidelines from the International Council on Harmonisation (2005) [38]. RPV and CAB standards were prepared in their respective mobile phase.

**Table VI:** HPLC–UV Parameters for Individual Quantification of RPV and CAB *In Vitro*

Analyte	Mobile phase	Flow rate	UV detection	Column temperature	Injection volume	Runtime	LoD (µg/mL)	LoQ (µg/mL)
RPV	Acetonitrile: 0.1% TFA (65:35 v/v)	1 mL/min	282 nm	30°C	50 μL	10 min	0.48	1.46
CAB	Acetonitrile: 0.1% TFA (90:10 v/v	0.8 mL/min	257 nm	40°C	50 μL	7 min	0.15	0.46

The simultaneous analysis of RPV and CAB from rat plasma samples was performed using an HPLC series system, the 1260 Infinity II Prime® (Agilent Technologies Inc.), coupled to a single quadrupole API 6400 mass spectrometer, also by Agilent Technologies, with electrospray ionisation in position ion mode to facilitate detection at lower concentrations. Mass spectrometry analysis was conducted in single-ion monitoring mode, with RPV and CAB detected at mass-to-charge ratios (m/z) of 367.4 and 406.3, respectively. The capillary voltage was established at 4 kV, drying gas temperature at 300°C, drying gas flow at 11 L/min, and the nebuliser pressure at 15 psi. Nitrogen was used as the source vapour and was maintained at 100 psi. The chromatographic separation was carried out by an Inertsil ODS-3 C18 column, (250 mm × 4.60 mm internal diameter, 5 μm packing). All analytical runs were preceded by a Phenomenex SecurityGuard™ HPLC cartridge of matching chemistry. The mobile phase consisted of 80:20 v/v acetonitrile and 0.1% TFA, at a flow rate of 0.5 mL/min. Column temperature was maintained at 40°C, and the injection volume was 5 μL. RPV peak detection occurred at 3.94 min, and CAB peak detection, at 6.07 min, with a total runtime of 10 min per sample to permit washout. Prior to use, mobile phases were degassed via filtration and sonication. Agilent OpenLAB® Software version 2.2. was used for chromatographic analysis. The LoD and LoQ for RPV were 18.47 ng/ml and 55.96 ng/ml, respectively, and for CAB, 75.25 ng/mL and 228.07 ng/mL, respectively. The HPLC–mass spectrometry analytical method was validated in accordance with the Q2(R1) guidelines from the International Council for Harmonisation (2005) [38]. RPV and CAB standards in plasma were prepared and extracted as detailed previously.

#### Statistical Analysis

All data were expressed as means ± standard deviation (SD) calculated using Microsoft Excel® 2016 (Microsoft Corporation, Redmond, Washington, United States). Statistical analysis was performed using GraphPad Prism® version 5.03 (GraphPad Software, San Diego, California, United States). Where appropriate, data were analysed by a paired *t-*test for comparison of two paired groups, an unpaired *t*-test was performed for comparison of two unpaired groups, and a one-way analysis of variance with post hoc Tukey test was used for comparison of multiple groups, with the data normally distributed. In all cases, a *p* value < 0.05 was denoted as a significant difference.

## Results and Discussion

### Formulation, Optimisation, and Post-Production Processing of ARV NS

The novel RPV NS prepared by the nanoprecipitation-ultrasonication process possessed a particle size of 203.54 ± 3.14 nm and a distribution of 0.18 ± 0.02. A negative surface charge (–11.74 ± 1.51 mV) resulted in electrostatic repulsion, thus minimising particle aggregation and promoting stability. Despite the use of a non-ionic stabiliser, Pluronic F-108, the formulation displayed a negative charge. This could be attributed to the anionic nature of RPV, which may adsorb to the nanocrystal surface, promoting this apparent surface charge [39]. The particle properties were found to be comparable to that of the Janssen proprietary formulation in the aqueous form, with less excipients [40]. RPV NS were lyophilised and subsequently reconstituted in deionised water, with the addition of mannitol 5% w/v, producing a mean particle size of 213.06 ± 2.59 nm and a PDI of 0.18 ± 0.01, exhibiting significant increases in neither diameter (*p* = 0.3260) nor dispersity (*p* = 0.4778) compared to respective properties pre-lyophilisation. In contrast, lyophilisation with no cryoprotectant resulted in significant increases in particle size to 252.32 ± 12.81 nm (*p* = 0.0014) and a less monodispersed NS with a PDI of 0.32 ± 0.06 (*p* = 0.0444). However, it was noted that, regardless of whether cryoprotectant was added or not, zeta potential remained unchanged, displaying a surface charge of –12.63 ± 1.08 mV with addition of mannitol 5% w/v following lyophilisation and –11.98 ± 1.25 mV without, neither of which was considered significant compared to the pre-lyophilised counterparts (*p* = 0.8313 and 0.2400, respectively). Consequently, mannitol 5% w/v was included in the formulation, both to avoid freeze damage due to ice formation and to further avoid particle aggregation [41]. The drug content of the lyophilised RPV NS was determined to be 70.6 ± 0.1% w/w of dry powder. This high drug concentration was necessary for incorporation into the dissolving MAP system requiring a high drug loading.

To maximise CAB loading per unit volume within the LA NS, the excipient containing supernatant was removed by needle and syringe, as described by a previous method [29], with reproducibility assured by measurement of mass loss. Removal of excipient containing supernatant was found not only to significantly increase drug concentration *(p* = 0.0006) but also to have no significant effect upon particle size (*p* = 0.0661), as shown in Table VII.

**Table VII:** Effect on Particle Properties and Drug Content Following Removal of Excipient-Containing Supernatant to Concentrate CAB Content per Unit Volume (Means ± SD, *n* = 3)

NS characteristic	Mean particle size (d. nm)	PDI	Zeta potential (mV)	Drug content (mg/mL)
Original CAB LA NS	340.68 ± 5.54	0.17 ± 0.05	–26.49 ± 1.51	193.41 ± 6.45
After removal of supernatant	344.80 ± 7.39	0.17 ± 0.06	–27.51 ± 1.43	390.26 ± 14.19

### Characterisation of Novel Lyophilised RPV NS

#### Determination of Physical and Chemical Interactions of Novel Lyophilised RPV NS

**Fig. 4:**
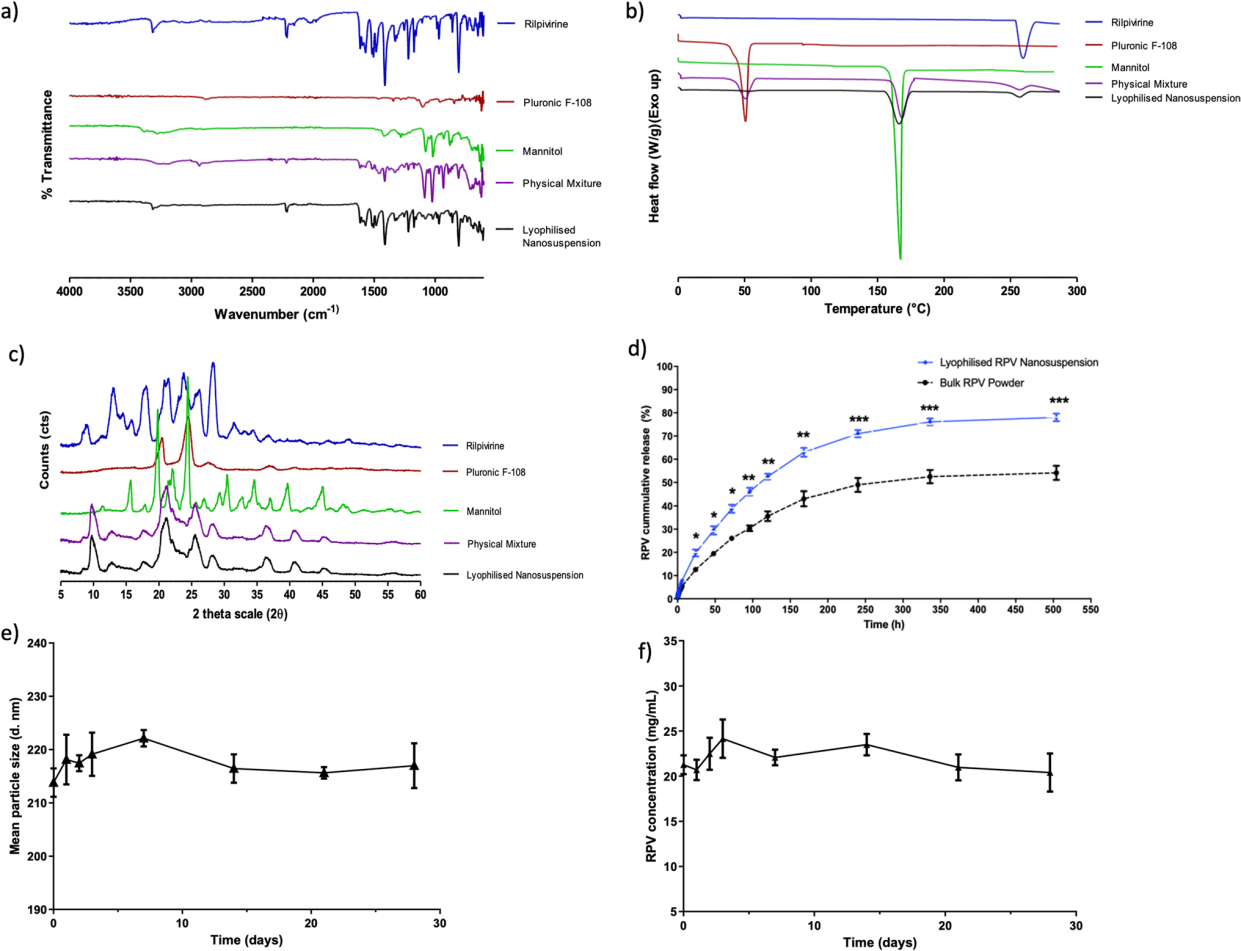
(**a**) ATR-FTIR spectra of RPV and NS excipients. (**b**) DSC thermograms of RPV and NS excipients. (**c**) P-XRD diffractograms of RPV and NS excipients. (**d**) In vitro release profiles of RPV in PBS (pH 7.4) / methanol (20/80% v/v) from bulk RPV and lyophilised RPV NS; *p* ≤ 0.033 (*), ≤ 0.002 (**), < 0.001 (***). (**e**, **f**) Evaluation of short-term stability of lyophilised RPV NS stored at room temperature (19°C) and protected from light, showing (**e**) mean particle size (d.nm) and (**f**) concentration (mg/mL) of RPV. (Means ± SD, *n* = 3).

Figure 4a illustrates the ATR-FTIR spectra of RPV, Pluronic F-108, mannitol, physical mixture, and lyophilised RPV NS. As displayed, the spectrum of RPV is characterised by typical absorption bands at approximately 3316 cm^−1^ (N–H), 2214 cm^−1^ (C≡N), 1651 cm^−1^ (C = O stretch), 1497 cm^−1^ (aromatic C = C), 1435 cm^−1^ (C-H bending), 1338 cm^−1^ (-CH wagging) and 1199 cm^−1^ (symmetric C-N stretching). Additional absorption bands are observed at 1596 cm^−1^, 1537 cm^−1^, 1504 cm^−1^, 1249 cm^−1^, 1214 cm^−1^, 1179 cm^−1^, 1152 cm^−1^, 1070 cm^−1^, 967 cm ^−1^, and 804 cm^−1^. Peak intensities were only partially reduced in both physical mixture and lyophilised RPV NS, indicating a minor physical interaction between the drug and carrier excipients of the formulation. However, no additional peaks were observed in the lyophilised RPV NS, indicating the absence of any chemical interaction between RPV and carrier excipients.

The thermograms of lyophilised RPV NS and base materials are illustrated in Fig. 4b. RPV exhibited an endothermic T_max_ of 259.46°C, corresponding to the melting point of the crystalline form. Following the melt at 259.46°C, decomposition was observed. Whilst the characteristic features of the RPV peak was retained in both the lyophilised NS and physical mixture, the T_max_ distinctive of RPV shifted to 256.98°C in both formulations, indicating a small but insignificant (*p* = 0.0870) physical interaction between the drug and carrier excipients, corresponding with the ATR-FTIR spectral study findings. However, no chemical interactions were noted, as Pluronic F-108 and mannitol retained their characteristic T_max_ peaks in the lyophilised NS and physical mixture at 51.05°C and 159.24°C, respectively. The thermogram also indicated that RPV remained in the crystal form in the lyophilised NS.

The P-XRD diffractograms confirmed that base materials RPV, Pluronic F-108, and mannitol were crystalline, as displayed in Fig. 4c. Determinations of this morphology was fundamental, as crystalline drug molecules have been previously reported to change to the amorphous form through NS formulation or resultant lyophilisation [42]. Whilst this is known to enhance solubility and dissolution, amorphous particles are less stable and prone to Ostwald ripening [43]. RPV exhibited distinctive sharp peaks at 2Ɵ values of 12.98°, 17.94°, 21.48°, 23.78°, and 28.24° and minor peaks at 8.36° and 26.20°. Pluronic F-108 exhibited two sharp peaks at 20.56° and 24.45°. Mannitol produced two major peaks at 19.82° and 24.42°, with smaller but distinctive peaks at 15.68°, 22.04°, 30.48°, 39.68°, and 44.98°. All distinctive peaks were retained in both the lyophilised NS and the physical mixture, although the lyophilised NS showed slight dampening, which further reinforces the findings from the previous ATR-FTIR and DSC characterisation studies indicating a slight physical interaction between drug and excipients. However, no new peaks appeared in the lyophilised NS diffractograms, nor did any disappear, again indicating the absence of drug-excipient chemical interactions.

Whilst RPV distinctive features were noticeably diminished in the final lyophilised product compared to its bulk counterpart, this can be explained by the relative quantities of drug in comparison to excipients. Prior to lyophilisation, RPV accounted for 0.018% w/v of the NS compared to mannitol’s 5% w/v, which subsequently demonstrated the largest peaks in the resultant characterisation traces. Small physical drug-excipient interaction was observed by all three characterisation techniques, but this is not unexpected, as surfactant has physically absorbed to the drug nuclei surface, with additional cryoprotectant interlinking bonds.

Lastly, the mean total water content of the lyophilised RPV NS formulation was calculated to be 1.54 ± 0.28% w/w, demonstrating the freeze-drying cycle was successful at removing water and residual DMSO solvent.

#### *In Vitro* RPV Drug Release Study

The in vitro release profile for bulk free-base RPV and lyophilised RPV NS are illustrated in Fig. 4d. Due to the intrinsic hydrophobicity of RPV, dissolution rate was expectedly slow, despite a high organic concentration of the dissolution media. However, formulation of RPV as a NS increased the rate of release and produced a greater cumulative release over the study period when compared to the pure drug. The increased rate of dissolution exhibited by the lyophilised RPV NS is a direct result of reduction of particle size, consequently increasing surface area, which leads to an enhanced dissolution rate and saturation solubility. Additionally, inclusion of mannitol within the formulation provides enhanced dissolution due to its disintegration properties [44]. Differences in the rate of release were noticeable after 4 h, with the NS showing significantly higher release from this point onward (*p* < 0.05). After 24 h, 19.75 ± 1.49% of RPV was released from the lyophilised NS, in comparison to 12.57 ± 0.20% of the bulk drug, displaying significantly increased release (*p* = 0.0012). Notably, neither form of RPV achieved close to 100% cumulative release; as such, final samples were taken at the 21-d time point, assuming equilibrium had been established. At the final sampling, lyophilised RPV NS achieved 78.03 ± 1.63% in contrast to 54.12 ± 3.07% of the pure drug, showing a significantly higher release profile (*p* = 0.0003). However, it must be noted that the dissolution rate exhibited in this permeation study by either the lyophilised or bulk RPV is unlikely to occur in the skin due to the volume of interstitial fluid being much lower than the volume of dissolution media and high levels of organic solvent (80% v/v methanol) in the dissolution media, unlike biological fluids. This was used solely for release comparison purposes; therefore, despite a prolonged release profile over three weeks, this may equate to even longer duration *in vivo.*

#### Evaluation of Novel Lyophilised RPV NS Short-Term Stability

The short-term stability of the novel lyophilised RPV NS was evaluated at room temperature (19°C), protected from light across a period of 28 d. Storage in the lyophilised NS form demonstrated no significant increases in particle size for the entire 28-d period (*p* = 0.0936) (Fig. 4e). Similarly, no significant changes in RPV concentration were established across the duration of the study (*p* = 0.5405) (Fig. 4f). Furthermore, no changes in physical appearance were noted throughout the entirety of the study, indicating that the lyophilised RPV NS is amenable to MAP manufacture at room temperature.

### Evaluation of the Effect of Polymer and MAP Formulation Upon Particle Size and PDI of ARV NS

As the ARV NS are situated within the dissolving polymeric matrix of the MAP system, consideration must be given throughout the fabrication process to the effect of formulation additives upon NS particle characteristics. Whilst expected that addition of polymer and excipients to the formulation will have some effect on particle properties, this effect should be minimised where possible so as not to detrimentally affect the inherent characteristics (i.e., particle size or surface charge) and stability (i.e., aggregation) of the NS system [20]. Accordingly, the mean particle size was evaluated before and after the formulation process for each of the ARV NS. In this study, PVA (9–10 kDa) and PVP (58 kDa) were selected as the MAP matrix formed as previously indicated in Tables I, II, and III.

For the lyophilised RPV NS, concentration-enhanced CAB LA NS and Janssen RPV LA NS, addition of PVA (9–10 kDa) demonstrated no significant increases in mean particle size (*p* > 0.05) nor affected PDI (*p* > 0.05). Furthermore, no significant changes in mean particle size (*p* > 0.05) or PDI (*p* > 0.05) were noticed upon addition of PVA (9–10 kDa), followed by slow addition of low concentrations of PVP (58 kDa). As such, compositions of these two polymeric blends were used in the MAP formulation for both MAP designs and ARVs.

**Fig. 5:**
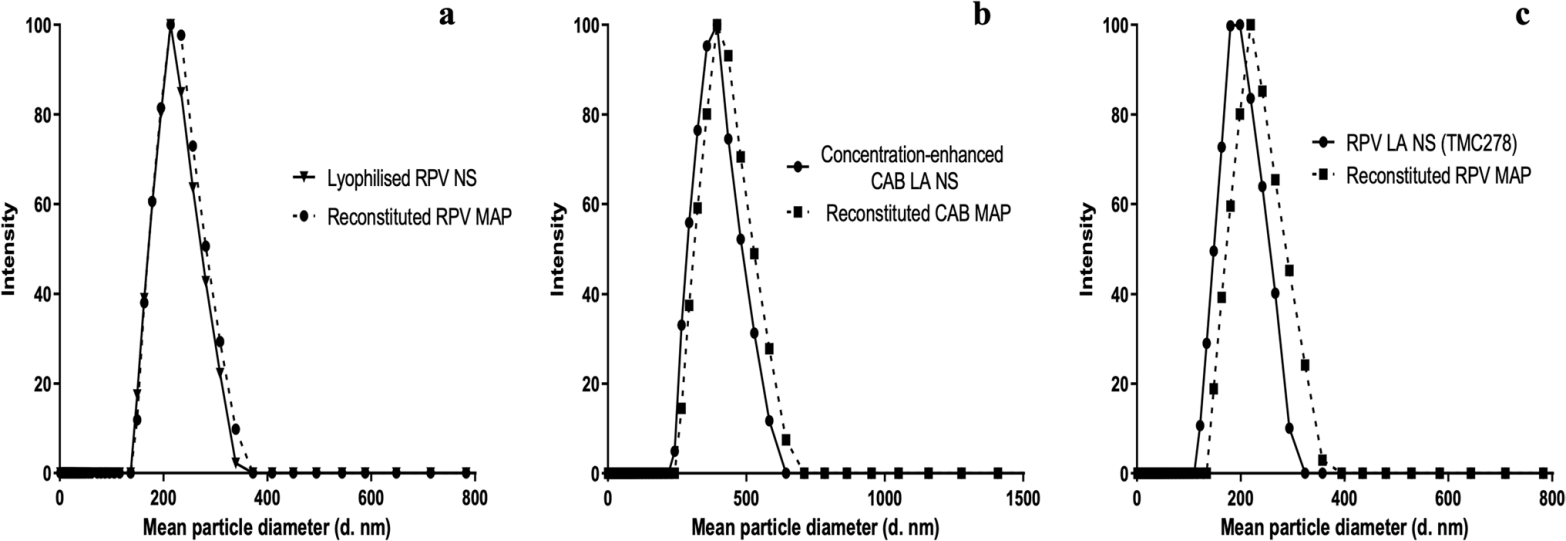
Comparison of the mean particle sizes of (**a**) novel lyophilised RPV NS, (**b**) concentration-enhanced CAB LA NS, and (**c**) RPV LA NS, to mean particle sizes of the NS following reconstitution of the MAP in deionised water. Reconstituted ARV MAPs representative of final ARV MAP formulations in each case.

Importantly, upon final formulation and optimisation of each ARV in the two MAP designs, the MAPs were reconstituted in water, and the mean particle size was compared to the original nanoparticle characteristics. In each case, no significant increase in particle size was established (*p* > 0.05) nor change in PDI (*p* > 0.05), suggesting there was no alteration of the original particle characteristics upon MAP formulation (Fig. 5).

### Fabrication of Two-Layered Dissolving 19 × 19 MAPs Containing ARV NS

**Fig. 6:**
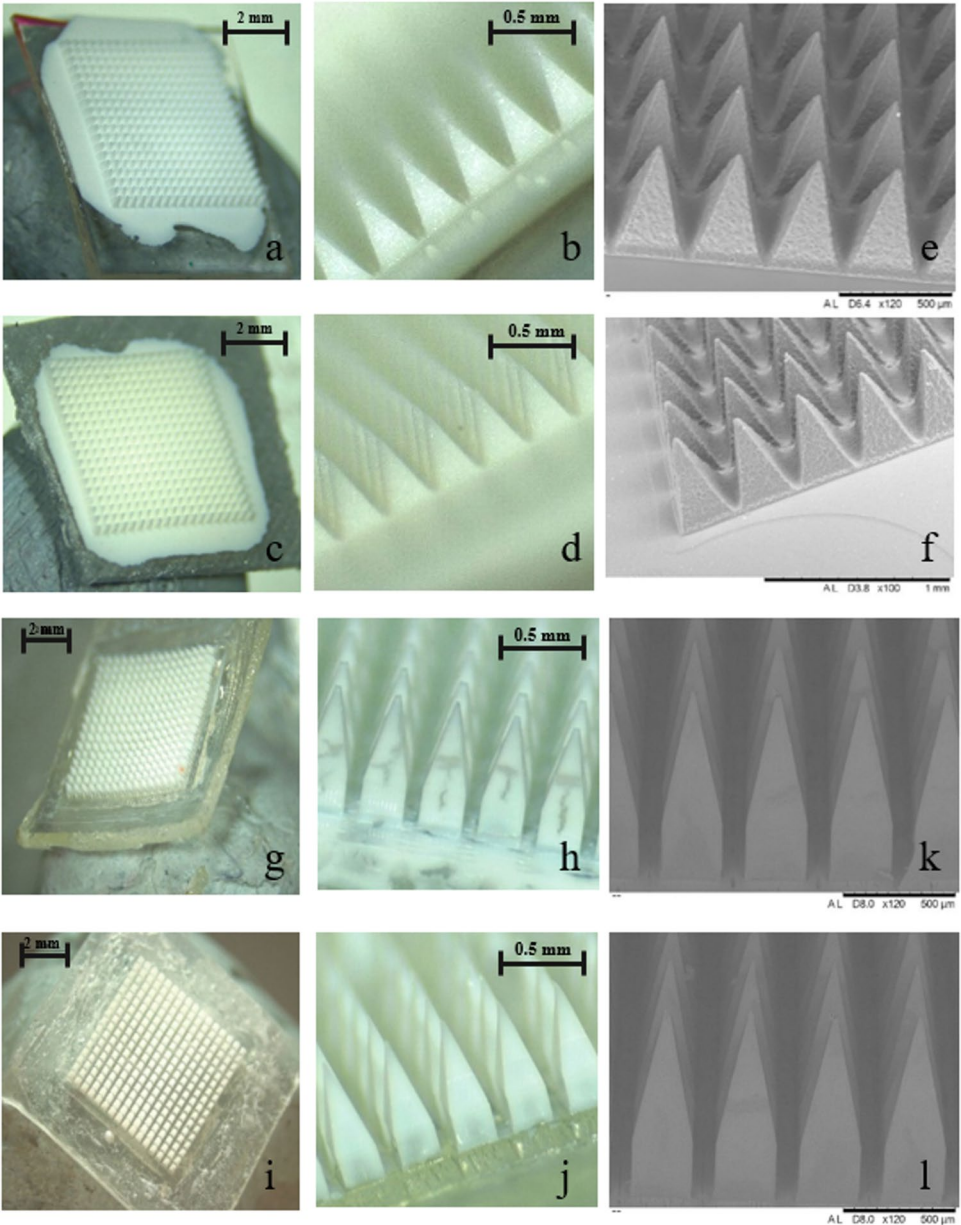
Light microscope images of optimised formulations representative of RPV 1.2 19 × 19 RPV MAPs **(a, b)**, CAB 1.6 19 × 19 CAB MAPs **(c, d)**. SEM images of 19 × 19 RPV MAPS **(e)** and 19 × 19 CAB MAPs **(f)** representative of the same optimised 19 × 19 formulations. Light microscope images of optimised formulations representative of RPV 2.3 16 × 16 RPV MAPs **(g, h)**, CAB 2.2 16 × 16 CAB MAPs **(i, j)**. SEM images of 16 × 16 RPV MAPS **(k)** and 16 × 16 CAB MAPs **(l)** representative of the same optimised 16 × 16 formulations.

As indicated by Tables I and II for RPV and CAB, respectively, PVA (9–10 kDa) and PVP (58 kDa) were used in the dissolving 19 × 19 MAP matrix formulation. Additionally, concentrations of lyophilised RPV NS or concentration-enhanced CAB LA NS were optimised to achieve the highest potential drug loading for the system. The formulation and timing of the baseplate addition to the MAPs were previously optimised (data not shown) and dependent upon the viscosity of the aqueous blend and the resultant drying time. The addition of a secondary baseplate has been shown to increase mechanical properties and insertion capabilities of the MAPs, whilst simultaneously reducing drug wastage through localisation of the drug solely in the needle tips [27]. Observations of the MAPs showed that prepared RPV and CAB 19 × 19 MAPs exhibited homogenous polymer blends, with the resultant MAPs having sharp needle tips. The morphology of all MAPs was examined using a stereomicroscope, and representative images of optimised MAP formulations of RPV 1.2 and CAB 1.6 are displayed in Fig. 6a–d. Moreover, representative SEM of images of the same respective formulations for RPV and CAB 19 × 19 MAPs are presented in Fig. 6e and f.

### Fabrication of Bilayer Dissolving 16 × 16 MAPs Containing ARV NS

The 16 × 16 bilayered MAPs were manufactured with the drug contained in the pyramidal needle tips and with a rapidly dissolving polymeric columnar base shaft. A polymeric baseplate was added in the aqueous form to enhance the rigidity of the MAP upon skin insertion. This approach was used to enhance skin insertion of ARVs, as it has been previously shown that only drugs that penetrate beneath the skin are readily available to be absorbed systemically and that any drug on the surface will be lost [27]. Similar to the 19 × 19 MAPs manufacture, aqueous blends of biocompatible polymers of PVA (9–10 kDa) and PVP (58 kDa) were used in the dissolving 16 × 16 MAP matrix formulation (Table III). Additionally, different concentrations of lyophilised RPV NS, aqueous Janssen RPV LA NS, or concentration-enhanced CAB LA NS were used to achieve the highest potential drug loading for the system. The previous RPV MAP formulation employed in the production of the pyramidal 19 × 19 MAPs, which used the lyophilised RPV NS, failed to produce 16 × 16 MAPs. This was in part due to the high viscosity of the polymer-NS blend, which is not capable of adequately filling the longer (850 µm) needle shafts, despite the application of an increased positive pressure. It was deemed necessary to use RPV LA NS in the 16 × 16 MAP formulation, as they were less viscous in the aqueous form and have been previously used in successful MAP formulation [27]. This would also explain the differences in RPV content within the 19 × 19 1.2 RPV MAP and 16 × 16 2.3 RPV MAP systems. It was shown that a reduction in ARV drug concentration, and conversely an increase in PVP (58 kDa) concentration, resulted in the most promising formulations upon casting formulations RPV 2.3 and CAB 2.2, respectively.

The morphology of MAPs was examined using a stereomicroscope, and representative images of optimised MAP formulations of RPV 2.3 and CAB 2.2 are displayed in Fig. 6g–i. Moreover, representative SEM images of the same respective formulations for RPV and CAB 16 × 16 MAPs are presented in Fig. 6j and k. Interestingly, upon microscopic examination of the ARV MAPs, CAB demonstrated homogenous dispersion throughout the MAP tips; however, RPV displayed distinct separation from the polymer, distributing towards the outer surface of the MAP tip and leaving a central clearing of assumedly drug-free polymer. This may be attributable to the hydrophobicity of the silicone micromould, to which hydrophobic RPV would inherently migrate, (Fig. 6h). Consequently, future work requires reduction of the surface hydrophobicity of the silicone moulds, utilising processes such as cold-plasma pretreatment, previously employed in the production of plastics [45].

### Characterisation of Mechanical and Insertion Properties of ARV MAPs

**Fig. 7:**
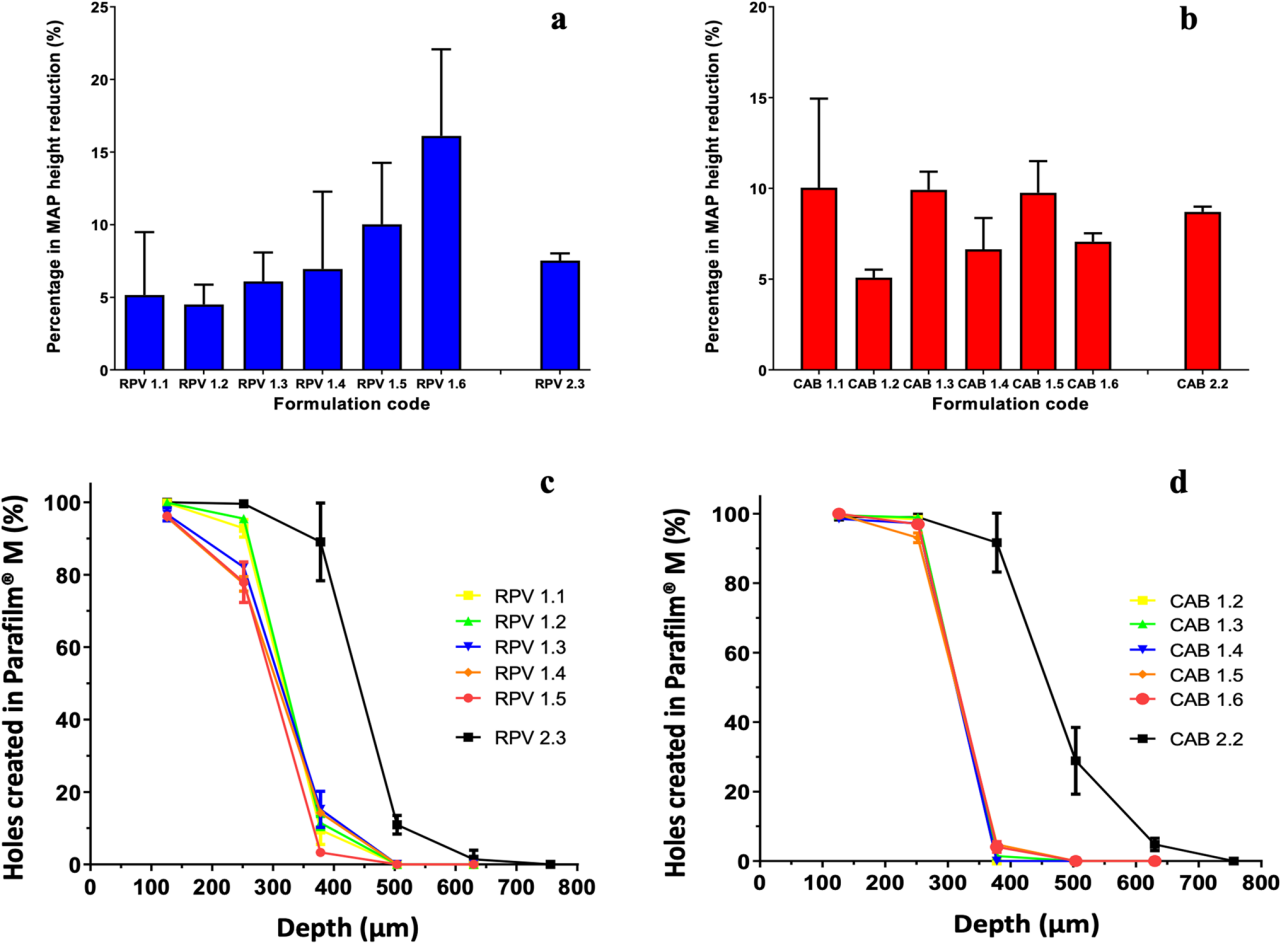
(**a**, **b**) Comparison of percentage height reduction of ARV MAPs following compression of 32 N/array, for (**a**) 19 × 19 and 16 × 16 RPV MAPs and (**b**) 19 × 19 and 16 × 16 CAB MAPs. (Means + SD, *n* = 5). (**c**, **d**) Percentage of holes created in Parafilm® M layers by ARV MAPs following an insertion force of 32 N/array, for (**c**) 19 × 19 and 16 × 16 RPV MAPs and (**d**) 19 × 19 and 16 × 16 CAB MAPs. (Means + SD, *n* = 3).

The success of a dissolving MAP system to achieve therapeutic delivery depends on its ability to penetrate the stratum corneum; as such, it must possess sufficient mechanical strength to do so upon application to the skin. Consequently, dissolving ARV MAPs were subjected to various mechanical characterisation tests. Initially, to narrow formulation selection, MAPs were subjected to evaluation of their mechanical strength in vitro [46]*.* Whilst a height reduction of < 5% is generally desirable, this is often unobtainable within dissolving MAP matrices requiring a substantially high drug loading, particularly of crystalline nature. As such, 19 × 19 RPV MAPs that displayed between 5 and 10% height reduction following compression by a 32 N force per array were advanced into further mechanical characterisation. Formulations that comprised relatively equal amounts of drug to polymer resulted in strong robust tips. In contrast, those with higher drug loading fractured upon compression and were deemed unsuitable for skin application. As illustrated by Fig. 7a, all 19 × 19 RPV MAP heights reduced following compression by a 32 N force per array; however, only RPV 1.6 was statistically different (*p* < 0.05) and was therefore not subjected to further characterisation testing.

Similar to 19 × 19 RPV MAPs, 19 × 19 CAB MAPs displayed between 5 and 10% height reduction in the needle tips (Fig. 7b), following compression by the same force. No significant differences in the reduction of 19 × 19 CAB MAP needle height was observed between the formulation which displayed the lowest percentage height reduction, namely CAB 1.2 (5.08 ± 0.44% height reduction), and that of the other CAB 19 × 19 formulations (*p* > 0.05). As such, all formulations were subjected to further characterisation testing. Evaluation of each of the selected 16 × 16 ARV MAP mechanical strength suggested that both RPV and CAB MAPs were robust (< 10% height reduction) (Fig. 7a and b).

Further mechanical characterisation evaluated the insertion capabilities of the dissolving ARV MAP using Parafilm M as an in vitro skin model, providing a cost-effective alternative and, additionally, avoiding dissolution of the needles in biological tissues for evaluation purposes [32]. The mean thickness of a Parafilm M layer is 126 ± 7 µm. The MAP insertion studies conducted suggest that all 19 × 19 ARV MAPs reside within the 2^nd^ and 3^rd^ layer of the skin simulant model. Considering the total height of the MAP tips was ~ 500 µm, this is representative of 252–378 µm of the tip insertion, equivalent to 50.4% to 75.6% of the total height (Fig. 7c and d). Similarly, for the 16 × 16 ARV MAPs, using Parafilm M suggested that both dissolving 16 × 16 ARV MAPs had been successfully inserted in vitro. Both 16 × 16 ARVs MAPs penetrate, at most, the 4^th^ layer of Parafilm M, equivalent to 59.3% of the total 850 µm MAP tip height (Fig. 7c and d). This is in line with previous insertion characterisation testing that demonstrates that 60% insertion achieved in vitro correlates to successful insertion into skin tissue *in vivo* [31, 32].

**Fig. 8:**
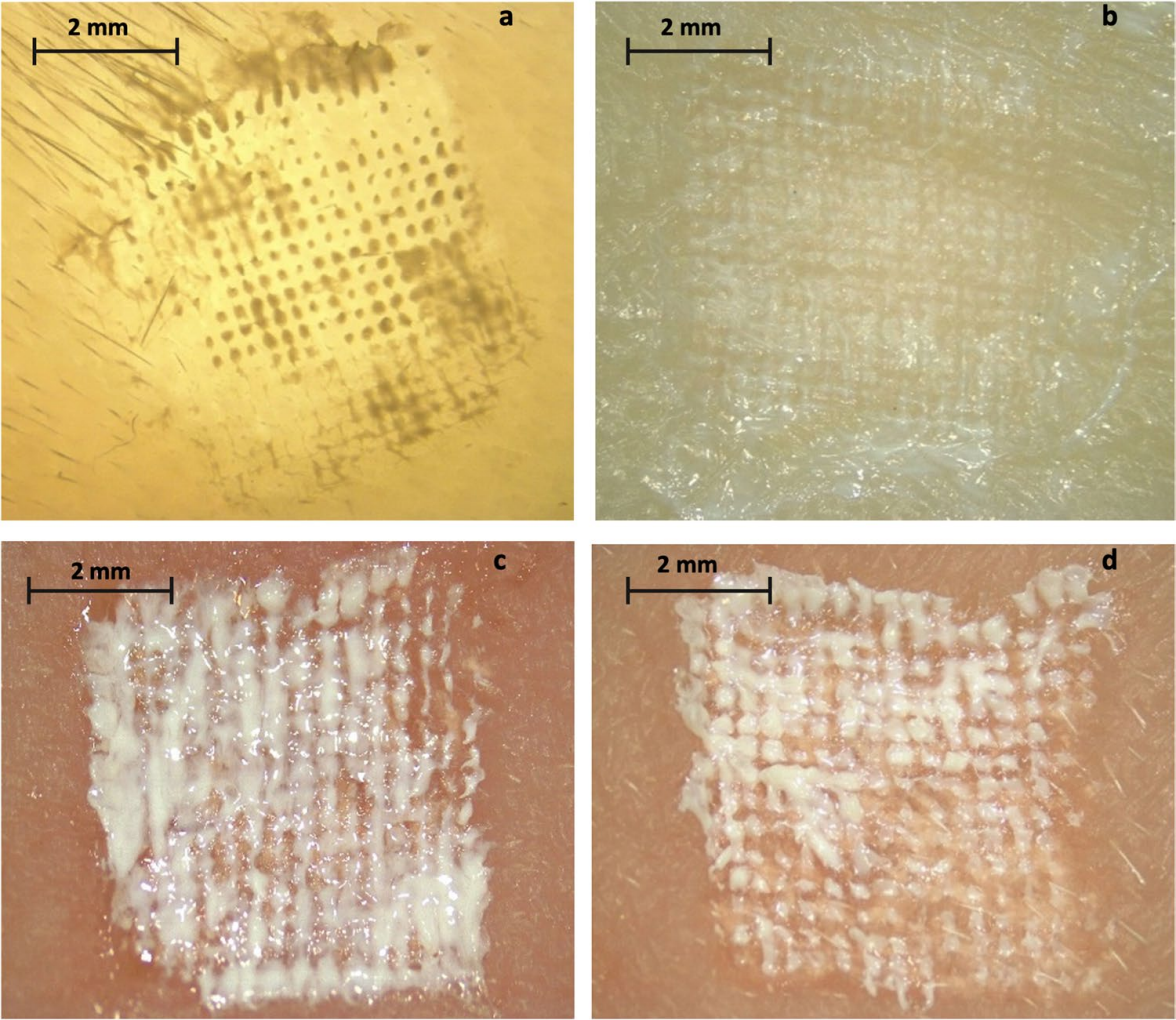
Light microscope images of excised neonatal porcine skin following insertion and removal of dissolving ARV MAPs. Displaying implantation of MAP tips in the skin for (**a**) 19 × 19 RPV MAP, (**b**) 19 × 19 CAB MAP, (**c**) 16 × 16 RPV MAP, and (**d**) 16 × 16 CAB MAP (*t* = 24 h).

Dissolving ARV MAPs were also manually inserted into excised full-thickness neonatal porcine skin and visually inspected by stereomicroscope at predetermined time points. Dissolution of 19 × 19 CAB MAPs was quicker than RPV 19 × 19 MAPs, with visible dissolution of 19 × 19 CAB MAPs apparent after 1 min and complete dissolution occurring within 20 min. For 16 × 16 RPV MAPs, visible dissolution occurred at 10 min and full dissolution at 25 min. Both visible and full dissolution was quicker than that observed with 19 × 19 RPV MAPs. For 16 × 16 CAB MAPs visible dissolution was at 1 min, achieving full dissolution at 20 min, comparable to the 19 × 19 CAB MAPs. Moreover, both ARV MAP designs displayed considerable tip implantation within the skin (Fig. 8a–d.) At this stage, the optimised formulations to be carried forward into more comprehensive testing were selected according to the shortest dissolution time and maximum drug loading. Therefore, RPV 1.2 and CAB 1.6 for the 19 × 19 MAP design were down-selected, in addition to RPV 2.3 and CAB 2.2 for the 16 × 16 MAP design.

### Evaluation of ARV Drug Content Localised Within MAP Tips

Using mathematical modelling, the theoretical drug content for the 19 × 19 and 16 × 16 ARV MAPs was determined (Table VIII). 19 × 19 1.6 CAB and 16 × 16 2.2 CAB MAPs contained 2.81 ± 0.43 mg and 2.86 ± 0.54 mg of CAB, respectively. 19 × 19 1.2 RPV and 16 × 16 2.3 RPV MAPs contained 1.73 ± 0.08 mg and 2.24 ± 0.35 mg of RPV, respectively. As mentioned previously, the differences in drug loading are attributed to the viscosity changes as a result of the two different RPV NS used.

**Table VIII:** The Mean MAP Drug Content for Each ARV and Respective MAP Design (Means ± SD, *n* = 5)

ARV MAP design	MAP drug content (mg)
19 × 19 1.2 RPV MAP	1.73 ± 0.08
19 × 19 1.6 CAB MAP	2.81 ± 0.43
16 × 16 2.3 RPV MAP	2.24 ± 0.35
16 × 16 2.2 CAB MAP	2.86 ± 0.54

### *Ex Vivo* Drug Deposition Studies

**Fig. 9:**
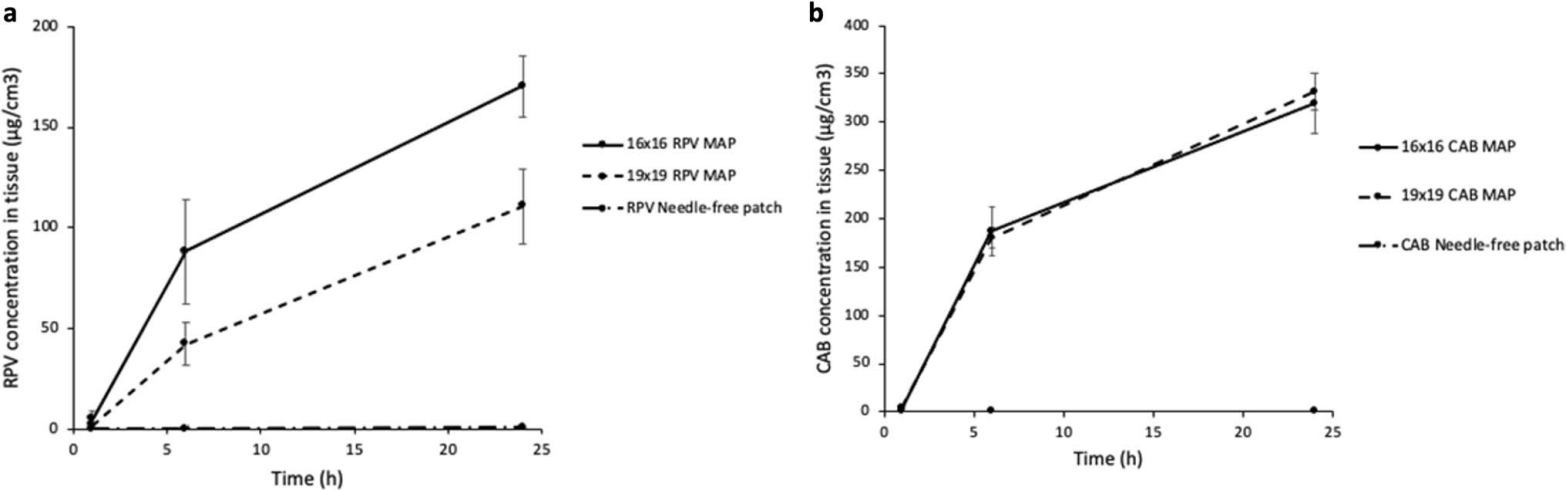
Concentration of (**a**) RPV and (**b**) CAB in full-thickness excised neonatal porcine skin following application of 19 × 19 and 16 × 16 MAPs inserted for three different durations (1, 5, and 24 h) and a needle-free patch as a control. RPV and CAB concentrations in excised neonatal porcine skin were determined at the respective time points and expressed as μg/cm^3^. This experiment was conducted under temperature control at 33°C (means ± SD, *n* = 3).

The amount of ARV deposited within the porcine tissue was quantified at three different application times – namely, 1, 5, and 24 h. The concentration of RPV and CAB in the tissue increased a function of the application time, with more drug deposited in the dermal layers the longer the MAP remained in place in the skin, as shown by Fig. 9a and b. The two RPV MAPs displayed marked increases in concentration at the 5- and 24-h time points, with 42.34 ± 10.77 µg/cm^3^ and 110.62 ± 18.63 µg/cm^3^ for the 19 × 19 1.2 RPV MAPs, and 88.12 ± 25.81 µg/cm^3^ and 170.26 ± 15.25 µg/cm^3^ for the 16 × 16 2.3 RPV MAPs, deposited after each time point, respectively. The two CAB MAPs displayed similar trends; however, with more drug deposited, owing in part to the less hydrophobic nature of CAB, 186.77 ± 25.50 µg/cm^3^ and 319.42 ± 31.47 µg/cm^3^ were deposited for the 16 × 16 CAB MAPs at the same respective time points. For both ARVs, the bilayer MAPs deposited more drug at each time point, owing to their greater drug load and faster dissolution. The 16 × 16 RPV MAPs deposited significantly more RPV at the 24-h time point than that of the 19 × 19 RPV MAPs (*p* = 0.0127); however, no significant difference in drug deposition was noted between the 19 × 19 and 16 × 16 CAB MAP at the 24-h time point (*p* = 0.8008). RPV and CAB both displayed significant increases in drug deposited within skin tissue between each successive MAP application time point (*p* < 0.0001), and similarly, a significant amount of drug was deposited after 24 h from a dissolving MAP in comparison to the needle-free control for both ARV compounds (*p* < 0.0001). The ARV needle-free film used as a control demonstrated negligible drug deposition in the skin tissue after 24-h application (< 1%) for each ARV compound of either MAP design. The relatively high amount of drug deposited in the dermal tissue by both MAP designs could result hypothetically in successful release *in vivo*.

### *In Vivo* Studies

#### Evaluation of Plasma Pharmacokinetics of RPV and CAB Following a Single MAP Application

This initial *in vivo* experiment assessed the pharmacokinetics of the two ARVs over an 84-d period following a single-dose application and was designed as a proof-of-concept study to explore a new route for sustained ARV delivery. As such, a four-way cohort comparison study was undertaken, dissolving 19 × 19 and 16 × 16 ARV MAPs and using IM and ID controls. Of the two control cohorts, IM administration of LA ARV NS was selected as the ‘true’ control to reflect the current method of administration of LA formulations. An ID injection was used to provide insights into the pharmacokinetics of ARV NS following administration to dermal tissue, as this is more representative of MAP-mediated ARV delivery.

At the site of application, visible implantation of the dissolved MAP tips containing the white-coloured ARV NS could be observed for both MAP cohorts following the removal of the film adhesive dressing after 24 h. Encouragingly, all the needles within the ARV MAPs displayed complete dissolution, with the exception of one 19 × 19 RPV MAP that left residual ‘stumps’, which is not surprising due to the highly hydrophobic drug content of the tips and which is consistent with the results exhibited *ex vivo* and with those of preliminary RPV work from within our research group [27].

**Fig. 10:**
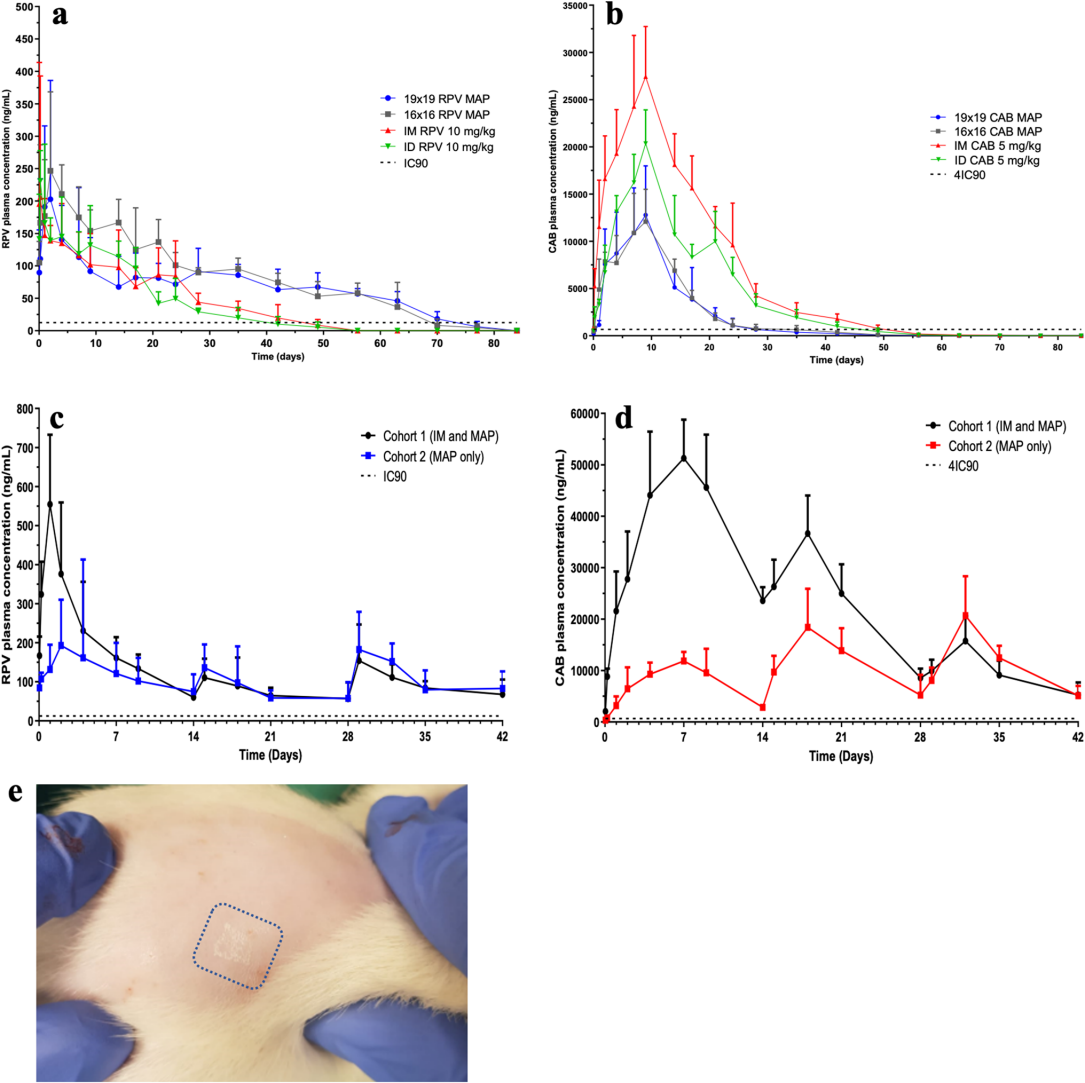
(**a**, **b**) The mean plasma concentrations and pharmacokinetic profiles of (**a**) RPV and (**b**) CAB in Sprague–Dawley rats following a single-dose application (means + SD, *n* = 6). (**c**, **d**) The mean plasma concentrations and pharmacokinetic profiles of (**c**) RPV and (**d**) CAB in Sprague–Dawley rats following administration of LA NS as an IM loading dose, followed by two repeated dissolving 16 × 16 MAP dose applications at 14-d intervals (cohort 1), or a dissolving 16 × 16 MAP loading dose, followed by two repeated dissolving 16 × 16 MAP dose applications at 14-d intervals only (cohort 2) (means + SD, *n* = 3 at 1 h, 5 h; *n* = 6 at all other sampling time points). (**a**–**d**) The IC_90_ (12 ng/mL) for RPV and 4xIC_90_ (664 ng/mL) for CAB is highlighted by the dashed black line. (**e**) Digital image displaying the dissolving 16 × 16 RPV MAP tips still implanted within the back of the rat’s skin two weeks following MAP application and removal (representative of *t* = 14 d).

Pharmacokinetic profiles of RPV and CAB after single-dose administration are displayed in Fig. 10a and b. Regarding RPV, both 19 × 19 and 16 × 16 dissolving RPV MAPs were capable of achieving therapeutically relevant concentrations above the currently advocated IC_90_ of 12 ng/mL [36] as soon as 1 h following application, and the concentrations continued to rise to their respective C_max_ values of 202.72 ± 183.40 ng/mL and 246.73 ± 121.62 ng/mL at a T_max_ of 2 d. Therapeutically relevant concentrations were maintained at Day 63 for both MAP conformations, at 45.99 ± 1427 ng/mL and 36.65 ± 37.84 ng/mL for the 19 × 19 and 16 × 16 RPV MAPs, respectively, but were below the system’s LoD at Day 70. As expected, IM and ID controls reached their C_max_ of 205.78 ± 187.16 ng/mL and 230.87 ± 47.00 ng/mL, respectively, much faster than that of both MAPs, with a T_max_ of 5 h. Nonetheless, no significant difference was noted between the C_max_ of the four RPV cohorts (*p* = 0.9451), with further post hoc analysis confirming no significant differences between any of the individual cohorts C_max_ (*p* > 0.05). This is most promising between the two RPV MAP cohorts, as whilst not directly comparable, they nevertheless demonstrate that the novel lyophilised RPV NS in the 19 × 19 MAPs can provide comparable release to that of the Janssen counterpart.

The pharmacokinetic results obtained following IM administration were comparable to those observed in previous studies using rats as an animal model. Van’t Klooster *et al*.(2010) [47] administered RPV LA to rats at a dose of 5 mg/kg, exhibiting a C_max_ of 71 ng/mL at a T_max_ of 7 h, in close agreement to that presently achieved. IM and ID controls both demonstrated prolonged release profiles following administration, with mean plasma concentrations of 34.44 ± 11.18 ng/mL and 29.26 ± 5.50 ng/mL at 35 and 28 d, respectively. Following a relatively high initial mean plasma concentration from both MAP conformations, concentrations declined throughout the remainder of the experiment, which extended further than the IM and ID controls. This was not unexpected, as solubilised RPV within the NS that entered the systemic circulation would account for the immediate high plasma concentrations that were detected. However, as time progressed the insoluble RPV nanocrystals deposited within the dermal tissue would gradually dissolve and reach systemic circulation. This theory was further supported by visible RPV implantation still present 14 d post-MAP application (Fig. 10e). In the only other study of its kind to deliver RPV LA via four dissolving 14 × 14 MAPs (600 µm in height and 300 µm interspacing) to a rat model *in vivo*, McCrudden *et al.* (2018) [27]demonstrated similar high initial mean plasma concentrations of RPV, proceeded by a fluctuated decline, providing further support of this theory. Further in line with the study conducted by McCrudden *et al.* (2018) [27], at the 56-d time point sampling, mean plasma concentrations of RPV were higher in rats that received MAP treatment than in those that received an IM injection. Owing to the extended-release profile, the AUC_t0–84_ obtained for the two RPV MAP cohorts in this study were deemed to be statistically higher than that of the IM and ID controls (*p* < 0.05). Further post hoc analysis confirmed significant pair-wise differences between the AUC_t0–84_ from all four cohorts, except between the AUC_t0–84_ for the control cohort that received RPV LA by IM injection and the AUC_t0–84_ for the control cohort that received RPV LA by ID injection (*p* > 0.05).

With respect to CAB, 19 × 19 and 16 × 16 dissolving MAPs were capable of achieving therapeutically relevant concentrations above the currently advocated 4xIC_90_ of 664 ng/mL [36] as soon as 24 h and 5 h following application, respectively. The more rapid 4xIC_90_ concentration obtained by the 16 × 16 MAP treatment cohort may be attributed to the longer MAP tip length’s depositing the CAB NS more closely to the dermal microcirculation, thus achieving faster systemic perfusion, which is further enhanced by faster solubilisation of the MAP tips. It must be noted, however, that due to Project Licence restrictions, no sampling took place between 5 and 24 h; therefore, it is likely that the 19 × 19 treatment cohort exhibited mean plasma levels above the 4xIC_90_ prior to the 24-h time point, as at 5 h a mean plasma concentration was displayed at 578.75 ± 390.34 ng/mL, just below the 4xIC_90_. The mean plasma concentrations then steadily increased to their C_max_ values of 12,786.00 ± 5199.52 ng/mL and 12,083.41 ± 3425.08 ng/mL for the 19 × 19 and 16 × 16 CAB MAPs, respectively, at the same T_max_ of 9 d. As was seen with RPV MAPs, the differences in C_max_ between both CAB MAP treatment cohorts were not deemed to be statistically significant (*p* > 0.05), demonstrating that they were comparable in terms of effectiveness of delivery.

The IM and ID controls achieved therapeutically relevant concentrations at 1 and 5 h, respectively. Interestingly, the ID control reached this level at the same time point as the 16 × 16 MAP. Mean plasma CAB levels then continued to rise to their C_max_ values of 27,423.86 ± 7534.50 ng/mL and 20,356.67 ± 3557.73 ng/mL for IM and ID, respectively, at the same T_max_ of 9 d. The differences in C_max_ values between IM and ID were not found to be of statistical significance (*p* > 0.05). Whilst limited studies exist on preclinical CAB pharmacokinetics, one such study performed by Jucker *et al.* (2017) [48] administered CAB LA to large rats (> 600 g) at a dose of 40 mg/kg. This resulted in a C_max_ of 70,530 ng/mL at a variable T_max_ ranging between 7 and 14 d. Accounting for differences in animal weight and dose administered, this is loosely comparable to that achieved within the *in vivo* study. IM and ID controls both demonstrated prolonged release profiles following administration, with mean plasma concentrations of 779.65 ± 345.48 ng/mL and 987.00 ± 472.74 ng/mL at 49 and 42 d for IM and ID, respectively, at which the following sampling time points were below the 4xIC_90_.

Analysis of the AUC_t0–84_ highlighted significant differences between the four CAB cohorts (*p* < 0.0001). Further post hoc analysis confirmed significant pair-wise differences between almost all AUC_t0–84_ from all four cohorts with the exceptions of the MAP treatment cohort that received 19 × 19 MAPs and the cohort that received 16 × 16 MAPs (*p* > 0.05), again reinforcing the comparable delivery capability of both MAP conformations.

Mean plasma concentrations of each ARV from both MAP conformations continued to rise after the 24-h MAP application period. Thus, RPV and CAB levels are being released from the ID micro-depots of solid-drug nanocrystals whilst concurrently being cleared from the body. This supports the theory that dissolving MAPs are simply a tool for administering the drug. Upon insertion into the skin, the micro-projections deposit the ‘true’ delivery system, the solid-drug NS, which then act to prolong release of the ARV.

The F of RPV and CAB in plasma after a single administration of 19 × 19 and 16 × 16 MAPs containing ARV NS compared to the IM administration of the LA ARV NS were calculated. Regarding RPV, the literature stipulates IM bioavailability (*F*_*IM*_) as 0.78 for rats [47]. The F of RPV from MAP with reference to IM injection was found to be 1.22 and 1.16 for the 19 × 19 and 16 × 16 RPV MAPs, respectively. The F of RPV from MAPs exhibited were superior to that of IM. This is a promising result, indicating that RPV MAPs have the potential to be used for disease management in those with HIV. Literature on CAB pharmacokinetics is extremely limited; as such, no *F*_*IM*_ in rats had been previously investigated. However, recent physiologically based pharmacokinetic modelling, generated from in silico data, suggests a cautious *F*_*IM*_ as 0.75 for rats [25]. In contrast to the unexpectedly high F of RPV, the F of CAB was calculated at 0.07 for both the 19 × 19 and 16 × 16 MAPs. Taking into consideration the estimated F of IM and MAP for CAB, it is clear that the IM administration was superior and warrants further optimisation of both MAP designs to respectively enhance the F of CAB via MAP-mediated administration.

Whilst pharmacokinetic parameters and plasma profile patterns generally were comparable between the dissolving 19 × 19 and 16 × 16 MAPs, highlighting the potential of the dissolving MAP platform for sustained ARV delivery, the 16 × 16 MAP design displayed slight superiority in the case of each ARV. Due to improved drug loading, the 16 × 16 MAPs were able to maintain therapeutically relevant concentrations for longer periods. Additionally, by minimising wastage of the expensive NS, these particular MAPs would be cheaper to manufacture.

#### Evaluation of Plasma Pharmacokinetics of RPV and CAB Following Multiple Repeated MAP Applications

The purpose of the second *in vivo* experiment was to ascertain the capability of repeated MAP applications to reproducibly achieve and maintain therapeutically relevant concentrations *in vivo*. Additionally, the study aimed to determine if an IM loading dose would have an impact upon the rate at which such concentrations were achieved. The IM loading-dose concentration of both ARV LA NS was increased twofold, based on the previous *in vivo* experiment, to determine if a higher dose would achieve the respective IC_90_ or 4xIC_90_ for RPV and CAB more rapidly. Pharmacokinetic profiles of RPV and CAB following multiple repeated dose administrations are displayed in Fig. 10c and d.

Regarding RPV, a therapeutically relevant concentration of RPV in plasma was achieved within 1 h for both cohorts 1 and 2, following which the mean plasma concentrations steadily increased to their respective C_max_ of 554.31 ± 178.3 ng/mL after 1 d for cohort 1 and to 193.05 ± 117.13 ng/mL after 2 d for cohort 2. Statistical analysis showed significant differences between the two initial C_max_ values (*p* = 0.0020). RPV mean plasma concentrations gradually declined after this time, prior to the second-dose application, to C_min_ values of 60.15 ± 19.51 ng/mL and 74.71 ± 44.34 ng/mL for cohorts 1 and 2, respectively, which were comparable (*p* = 0.4788). Following the MAP application at Day 14, mean plasma RPV concentrations gradually increased to C_max_ values of 110.49 ± 48.44 ng/mL and 135.85 ± 59.68 ng/mL at a T_max_ of 15 d (1 d post-application) for cohorts 1 and 2, respectively, which were insignificant between the two cohorts (*p* = 0.9373). The plasma concentrations once again gradually declined after this time, prior to the next MAP application, to C_min_ values of 57.00 ± 41.41 ng/mL and 57.70 ± 41.48 ng/mL for cohorts 1 and 2 at 28 d, respectively, which once again were comparable (*p* = 0.9862). Following the final MAP application, mean plasma concentrations gradually increased to C_max_ values of 154.38 ± 92.17 ng/mL and 182.59 ± 96.49 ng/mL at a T_max_ of 29 d (1 d post-application) for cohorts 1 and 2, respectively, showing no statistical significance between the two cohorts again (*p* = 0.6158). Following which mean plasma concentrations gradually declined until the final sampling point at 42 d, at which point the mean plasma concentrations were still above the IC_90_ and were comparable between the two cohorts (*p* = 0.5346). The AUC_t0–42_ for RPV treatment cohort 1 was 5176.39 ± 454.25 ng/mL. Cohort 2 had an AUC of 4003.52 ± 928.36 ng/mL. For both cohorts, therapeutically relevant plasma concentrations were maintained throughout the study duration.

With respect to CAB, a therapeutically relevant concentration of CAB in plasma was achieved within 1 h for cohort 1 and within 5 h for cohort 2. Following this, the mean plasma concentrations steadily increased to their respective C_max_ of 551,290.320 ± 7,499.07 ng/mL and 11,844.70 ± 1,783.77 ng/mL for cohorts 1 and 2, respectively, at the same T_max_ value of 7 d. Statistical analysis showed significant differences between the two initial C_max_ values (*p* < 0.0001). CAB mean plasma concentrations gradually declined after this time, prior to the second-dose application, to C_min_ values of 23,591.160 ± 2,611.44 ng/mL and 2,842.10 ± 315.53 ng/mL for cohorts 1 and 2, respectively, which again were significant (*p* < 0.0001).

Following the MAP application at Day 14, mean plasma CAB concentrations gradually increased to C_max_ values of 36,640.61 ± 7,394.25 ng/mL and 18,394.10 ± 7,519.13 ng/mL at a Tmax of 18 d (3 d post-application) for cohorts 1 and 2, respectively. Statistical analysis of the second C_max_ was found again to be significant between the two cohorts (*p* = 0.0017). The plasma concentrations once again gradually declined after this time, prior to the next MAP application, to C_min_ values of 8,558.14 ± 1,817.92 ng/mL and 5,229.61 ± 3,939.18 ng/mL at 28 d for cohorts 1 and 2, respectively, which at this stage were now comparable (*p* = 0.0888).

Following the final MAP application, mean plasma concentrations gradually increased to C_max_ values of 15,753.65 ± 4,815.91 ng/mL and 20,674.78 ± 7,649.27 ng/mL at a T_max_ of 32 d (4 d post-application) for cohorts 1 and 2, respectively. Encouragingly, statistical analysis of the final C_max_ showed no significance between the two cohorts (*p* = 0.2119). Following which mean plasma concentrations gradually declined until the final sampling point at 42 d, at which point they were still above the 4xIC_90_ and were comparable between the two cohorts (*p* = 0.0.9145). Cohort 1 had an AUC_t0–42_ of 986,247.29 ± 64,833.43 ng/mL. The AUC_t0–42_ for cohort 2 was 436,293.58 ± 77,908.48 ng/mL. Importantly, therapeutically relevant plasma concentrations were maintained throughout the study duration for both cohorts.

The second *in vivo* study demonstrated that therapeutically relevant concentrations were achieved rapidly for both ARVs, regardless of the nature of loading-dose administration. Additionally, following the second-dose application for RPV and third-dose application for CAB, plasma profiles and pharmacokinetic parameters were comparable. Promisingly, therapeutically relevant concentrations were maintained throughout the duration of the study for both ARVs, demonstrating the potential of the dissolving MAP platform as a delivery device to reproducibly achieve therapeutic outcomes. It should be noted that, whilst plasma profiles and pharmacokinetic parameters (i.e., C_max_) varied between cohort designs, the purpose of this *in vivo* experiment was not to achieve the highest maximal concentration but rather to achieve and maintain IC_90_ or 4xIC_90_ for RPV and CAB, respectively. Sampling was selected to capture the plasma profiles pre- and post-dose administration. Whilst this may not provide a complete pharmacokinetic picture representative of the true T_max_ or C_max_ values, the values obtained were consistent between the two cohorts, and as such considered valid for the purposes and scope of the research under investigation. Notably, as the IM loading dose of each ARV was increased twofold upon the initial *in vivo* study, a representative twofold increase in respective C_max_ for each ARV was demonstrated.

The dose administered and dosing interval must be selected not only to achieve the plasma therapeutic limits of each ARV but also to avoid toxicity and adverse drug reactions. Both dose and dosing interval are key to avoiding accumulation of the ARV in plasma in a ‘step-wise’ manner with each successive MAP application, which may lead to toxic effects. However, whilst further extensive pharmacokinetic analysis is undoubtedly required, the work presented here in this second *in vivo* study provides encouraging preliminary data, which may serve as a proof of concept, indicating the potential removal of the need for hypodermic needle and syringes in the future administration of LA ARVs to maintain viral suppression. Using MAP technology, avoidance of cold-chain storage may enable cost-effective development. This would increase suitability of treatment in low-resource settings by reducing associated treatment costs as well as removing the need of a trained HCP for administration purposes. Additional formulation optimisation, followed by pharmacokinetic evaluation in larger animal models which more closely mimic humans’ pharmacokinetic profiles, is now warranted to further investigate the claims from the *in vivo* studies conducted here.

## Conclusions

This proof-of-concept work outlines the formulation of two dissolving MAP systems for the sustained release of two LA ARV NS – namely, RPV and CAB. Comprehensive initial *in vivo* studies successfully reported therapeutically relevant delivery of RPV and CAB in Sprague–Dawley rats following a single MAP application for prolonged periods – specifically, up to 70 d and 63 d for the 19 × 19 and 16 × 16 RPV MAPs, respectively, and 24 d and 28 d for the 19 × 19 and 16 × 16 CAB MAPs, respectively. This confirmed the success of both dissolving ARV MAP conformations in terms of insertion and ability to achieve sustained release. Further *in vivo* studies demonstrated reproducible therapeutic plasma concentrations following repeated MAP applications at 14-d intervals that loosely correlated to that of repeat IM dosing schedules. This innovative delivery approach of combining NS and MAPs represents a substantial opportunity to overcome the current requirement for adherence to daily oral multi-drug ARV regimens and further circumvent some of the problems associated with hypodermic needle delivery of ARV LA NS. Thus, future use of MAPs for needle-free delivery of ARVs for the prevention and treatment of HIV infection, particularly in the paediatric population, deserves exploration. MAPs could enable treatment in low-resource settings by reducing associated treatment costs and by removing the need for a trained health care professional for administration. Further MAP development, such as enhancing MAP tip length and array density, is now required to improve drug loading within the MAP system, leading to a decrease in patch size. In addition, HIV challenge models and physiologically based pharmacokinetic modelling are now essential to ensure that the true potential of this innovative drug delivery platform is fully realised.

## Abbreviations

AIDS: Acquired immune deficiency syndrome
CAB: Cabotegravir
HIV: Human immunodeficiency virus
MAP: Microarray patch
RPV: Rilpivirine
